# IL-17–producing **γδ** T cells in the tumor microenvironment promote radioresistance in mice

**DOI:** 10.1172/JCI193945

**Published:** 2025-10-07

**Authors:** Yue Deng, Xixi Liu, Xiao Yang, Wenwen Wei, Jiacheng Wang, Zheng Yang, Yajie Sun, Yan Hu, Haibo Zhang, Yijun Wang, Zhanjie Zhang, Lu Wen, Fang Huang, Kunyu Yang, Chao Wan

**Affiliations:** 1 Cancer Center, Union Hospital, Tongji Medical College and; 2 Institute of Radiation Oncology, Union Hospital, Tongji Medical College, Huazhong University of Science and Technology, Wuhan, China.; 3 Hubei Key Laboratory of Precision Radiation Oncology, Wuhan, China.; 4 Key Laboratory of Biological Targeted Therapy (Huazhong University of Science and Technology), Ministry of Education, Wuhan, Hubei, China.; 5 Cancer Center, Department of Radiation Oncology, Zhejiang Provincial People’s Hospital (Affiliated People’s Hospital), Hangzhou Medical College, Hangzhou, Zhejiang, China.

**Keywords:** Immunology, Oncology, Cancer, Immunotherapy, Radiation therapy

## Abstract

The immunosuppressive tumor microenvironment (TME) drives radioresistance, but the role of γδ T cells in regulating radiosensitivity remains incompletely understood. In this study, we found that γδ T cell infiltration in the TME substantially increased after radiotherapy and contributed to radioresistance. Depletion of γδ T cells enhanced radiosensitivity. Single-cell RNA-seq revealed that γδ T cells in the postradiotherapy TME were characterized by the expression of *Zbtb16*, *Il23r*, and *Il17a*, and served as the primary source of IL-17A. These γδ T cells promoted radioresistance by recruiting myeloid-derived suppressor cells and suppressing T cell activation. Mechanistically, radiotherapy-induced tumor cell–derived microparticles containing dsDNA activated the cGAS-STING/NF-κB signaling pathway in macrophages, upregulating the expression of the chemokine CCL20, which was critical for γδ T cell recruitment. Targeting γδ T cells and IL-17A enhanced radiosensitivity and improved the efficacy of radiotherapy combined with anti-PD-1 immunotherapy, providing potential therapeutic strategies to overcome radioresistance.

## Abstract 2

Tumor cell-derived dsDNA-containing microparticles activate macrophage cGAS-STING pathway, inducing CCL20 to recruit IL-17-producing γδ T cells, driving radioresistance. Targeting this axis improves radiotherapy response.

## Graphical abstract

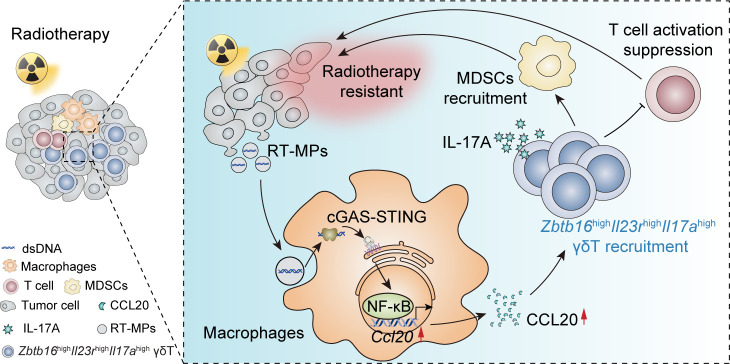

## Introduction

Radiotherapy is one of the primary therapeutic strategies for malignant tumors, with approximately 50% of cancer patients undergoing radiotherapy during the course of their disease (1, 2). Radiotherapy not only induces direct DNA damage to eradicate tumor cells, but also elicits antitumor immune responses through mechanisms such as the in situ vaccine effect and the “abscopal effect” (3, 4). However, emerging evidence highlights that, while radiotherapy activates antitumor immunity, it can also induce immunosuppressive effects (5). For example, increased glycolytic activity following radiotherapy leads to lactate accumulation and acidification of the tumor microenvironment (TME), which impairs the function of effector T cells, upregulates PD-1 expression on regulatory T cells (Tregs), enhances the tumor-promoting activity of myeloid-derived suppressor cells (MDSCs), and induces macrophage polarization toward the M2 phenotype (6–8). These changes ultimately undermine the tumor-suppressive effects of radiotherapy. Therefore, a deeper understanding of the interplay between radiotherapy and the tumor immune microenvironment (TIME) is crucial for optimizing radiotherapy treatment outcomes and harnessing the full potential of radiotherapy treatment to amplify antitumor immunity.

γδ T cells, characterized by their unique T cell receptor (TCR) composed of γ and δ chains, represent a distinct subset of T lymphocytes. In healthy adults, they typically constitute 1%–10% of peripheral blood T cells but are more abundant in mucosal tissues, such as the intestines and respiratory tract, as well as in subcutaneous tissues (9). Unlike conventional αβ T cells, which primarily recognize antigenic peptides presented by MHC molecules, γδ T cells can recognize and respond to nonclassical antigens expressed by tumor cells (10). This makes them a key component of the MHC-unrestricted innate-like T cell population. γδ T cells are capable of producing a wide array of bioactive factors, including IFN-γ, TNF-α, IL-17, and IL-4, which play critical roles in immune regulation and response (11). Current research on the functions of γδ T cells in antitumor immunity has identified 2 major subsets (12). One subset primarily secretes antitumor cytokines such as IFN-γ and TNF-α, enhancing the antitumor activity of NK cells, Th1 cells, and cytotoxic T lymphocytes (CTLs). The other subset predominantly secretes IL-17, which promotes the recruitment of MDSCs and Tregs, facilitates angiogenesis, and suppresses antitumor immunity (13). Despite their important roles in immune regulation and tumor surveillance, the impact of γδ T cells on radiotherapy sensitivity and their underlying mechanisms remain poorly elucidated.

Here, we demonstrate that the infiltration of γδ T cells in the TME is markedly increased after radiotherapy, which subsequently promotes radioresistance. Single-cell RNA-seq (scRNA-seq) analysis reveals that the γδ T cell population in the postradiotherapy TME is predominantly characterized by the expression of *Zbtb16*, *Il23r*, and *Il17a*, and serves as the major source of IL-17A in the TME. Functionally, these γδ T cells drive radioresistance by orchestrating the recruitment of MDSCs and suppressing T cell–mediated antitumor immunity. Mechanistically, we identify that radiated tumor cell–released microparticles (RT-MPs) containing double-stranded DNA (dsDNA) are taken up by macrophages. This process activates the cGAS-STING/NF-κB signaling axis in macrophages, leading to the upregulation of the chemokine CCL20, a critical mediator responsible for recruiting γδ T cells into the TME. Collectively, these findings unveil the important role and mechanisms of γδ T cells in regulating radiosensitivity, providing valuable insights for identifying therapeutic targets to overcome radioresistance.

## Results

### Enhanced γδ T cell infiltration in the TME following radiotherapy promotes radioresistance.

**Figure 1:**
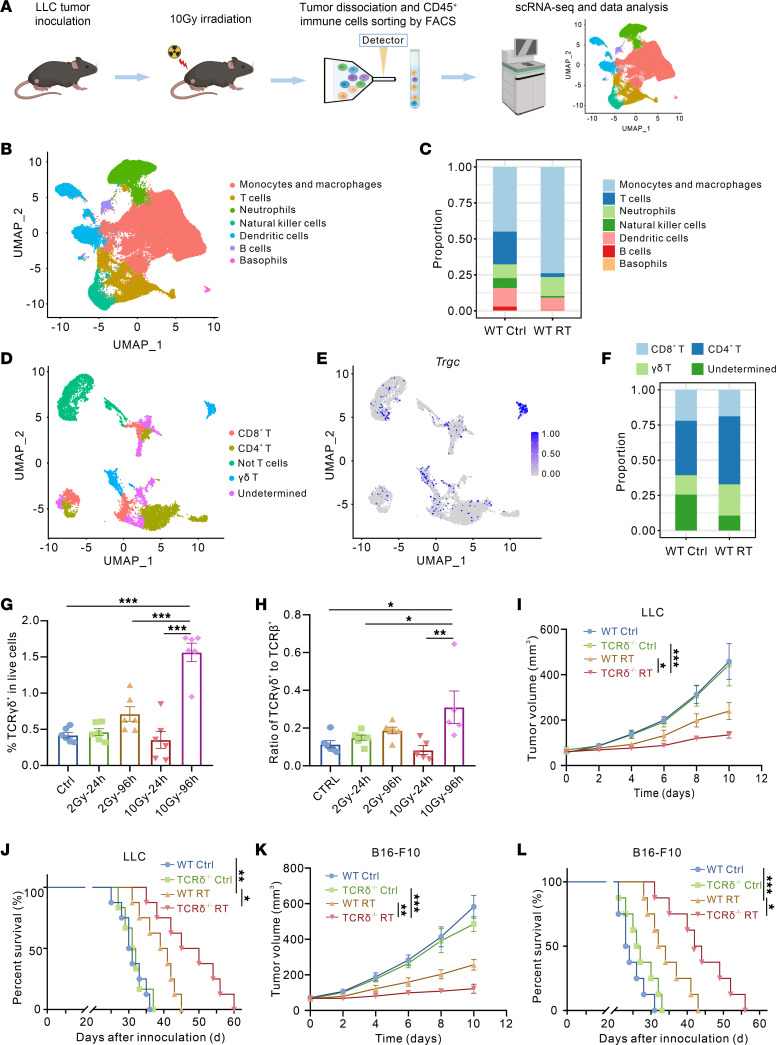
Radiotherapy-induced γδ T cell infiltration in the TME promotes radioresistance. (**A**) Schematic diagram for scRNA-seq of CD45^+^ immune cells isolated from mouse subcutaneous tumors. (**B**) UMAP plot of all cells passed quality control colored by cell identities. (**C**) Stacked bar plot showing the proportion of major immune cell types originating from WT Ctrl and WT RT mice. (**D**) UMAP plot of T cells colored by cell clusters as indicated. (**E**) Feature plots of the *Trgc* (referred to γδ T cells) expression in the T cell clusters. (**F**) Stacked bar plot showing the proportion of major T cell clusters originating from WT Ctrl and WT RT mice. (**G**) Flow cytometry analysis of γδ T cell proportions in the TME of LLC subcutaneous tumors following radiotherapy at different doses (2 Gy and 10 Gy) and time points (24 h and 96 h) (*n* = 6 per group). (**H**) Changes in the ratio of γδ T cells to αβ T cells in the TME following radiotherapy at different doses and time points (*n* = 5 to 6 per group). (**I**) Tumor growth curves of LLC subcutaneous tumors in corresponding groups (*n* = 6 to 8 per group). (**J**) Kaplan-Meier survival plot of LLC lung cancer–bearing mice in the corresponding groups (*n* = 6–8 per group). (**K**) Tumor growth curves of B16-F10 subcutaneous tumors in corresponding groups (*n* = 8 per group). (**L**) Kaplan-Meier survival plot of B16-F10 melanoma-bearing mice in the corresponding groups (*n* = 8 per group). *P < 0.05; **P < 0.01; ***P < 0.001. 1-way ANOVA with Tukey’s multiple comparisons test (**G** and **H**), 2-way ANOVA followed by Tukey’s multiple comparison test (**I** and **K**), Log-rank (Mantel-Cox) test (**J** and **L**).

To systematically delineate the alterations in immune cell composition within the TME after radiotherapy (RT), we conducted scRNA-seq on CD45^+^ immune cells isolated from murine Lewis lung cancer subcutaneous tumors in 2 groups: the control group and the RT group (96 hours after 10 Gy radiotherapy) (Figure 1A). Unsupervised clustering analysis identified 7 distinct immune cell subsets, including monocytes and macrophages, T cells, neutrophils, natural killer cells, dendritic cells, B cells, and basophils (Figure 1B and Supplemental Figure 1A; supplemental material available online with this article; https://doi.org/10.1172/JCI193945DS1). Comparative analysis revealed pronounced shifts in immune cell clusters following radiotherapy, characterized by increased monocyte and macrophage populations and decreased T cell and NK cell relative proportions, potentially attributed to the differential radiosensitivity between myeloid and lymphoid lineages (Figure 1C and Supplemental Figure 1B). To precisely characterize the dynamics of γδ T cells, we performed subclustering of the T cell cluster, delineating 5 distinct subsets including a clearly defined γδ T cell cluster (Figure 1, D and E). Notably, we observed a marked increase in the relative proportions of both CD4^+^ and γδ T cell subsets after radiotherapy (Figure 1F and Supplemental Figure 1C). To further validate these findings, we established the murine subcutaneous Lewis lung cancer model. Tumor tissues were collected at 24 hours and 96 hours after irradiation with a single-dose of 2 Gy or 10 Gy, followed by comprehensive T cell profiling using flow cytometry (Supplemental Figure 1D). Compared with lower-dose (2 Gy) radiotherapy or short-term (24 hours) postradiotherapy, the proportion of γδ T cell infiltration was markedly increased at 96 hours following higher-dose (10 Gy) radiotherapy (Figure 1G). Additionally, although αβ T cells exhibited increased proportions following radiotherapy at the same dose and time point, the γδ T/αβ T cell ratio showed the most substantial elevation, reflecting the predominant expansion of γδ T cells relative to αβ T cells postradiotherapy (Figure 1H and Supplemental Figure 1, E and F). These findings collectively demonstrated that radiotherapy induces preferential γδ T cell infiltration within the TME, warranting further investigation into their role in radiotherapy-mediated tumor immunity.

To investigate the functional role of γδ T cells in radiosensitivity, we established subcutaneous tumor models using TCR δ chain-deficient (TCR δ^–/–^) and age-matched WT mice. It has been confirmed that γδ T cells were genetically ablated in TCR δ^–/–^ mice, while αβ T cell populations remained intact. In Lewis lung cancer and B16-F10 melanoma subcutaneous tumor models, as well as orthotopic pancreatic tumor models, TCR δ^–/–^ mice exhibited notable tumor growth inhibition and prolonged overall survival compared with WT mice following single-dose 10 Gy irradiation (Figure 1, I–L, and Supplemental Figure 2A). This radiosensitization effect was further corroborated using the 8 Gy × 3 and 2 Gy × 5 regimens, demonstrating superior tumor growth control in TCR δ^–/–^ mice relative to WT mice (Supplemental Figure 2, B–E). These results highlight the critical role of γδ T cells in mediating tumor radioresistance.

### γδ T cells in the postradiation TME are characterized by IL-17 secretion.

**Figure 2:**
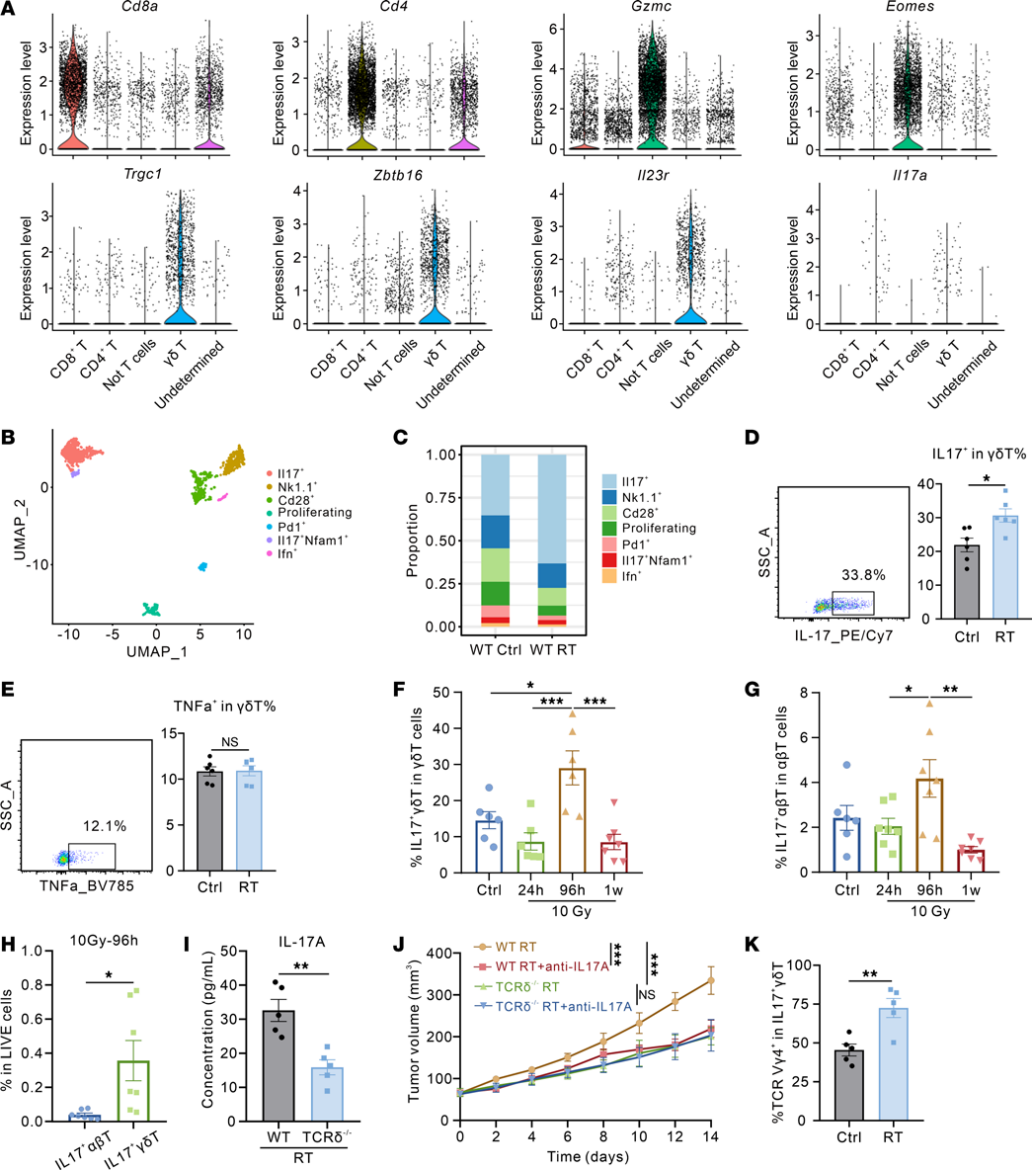
The γδ T cell population in the TME after radiotherapy is primarily characterized by the IL-17–producing subset. (**A**) Violin plots demonstrate expression of the genes that identify each T cell cluster. (**B**) UMAP plot of γδ T cells colored by cell clusters as indicated. (**C**) Stacked bar plot showing the proportion of major γδ T cells clusters originating from WT Ctrl and WT RT mice. (**D**) Representative flow cytometry plots and statistical analysis of IL-17 expression in γδ T cells from LLC subcutaneous tumors after radiotherapy (*n* = 6 per group). (**E**) Representative flow cytometry plots and statistical analysis of TNF-α expression in γδ T cells from LLC subcutaneous tumors after radiotherapy (*n* = 6 per group). (**F**) Flow cytometry analysis of IL-17^+^ γδ T cell proportions in the TME of LLC subcutaneous tumors following radiotherapy at different time points (*n* = 6–7 per group). (**G**) Flow cytometry analysis of IL-17^+^ αβ T cell proportions in the TME of LLC subcutaneous tumors following radiotherapy at different time points (*n* = 6–7 per group). (**H**) Proportions of IL-17^+^ γδ T cells and IL-17^+^ αβ T cells in the TME at 96 hours after 10 Gy radiotherapy (*n* = 7 per group). (**I**) IL-17A concentrations in tumor interstitial fluid from WT RT and TCRδ^–/–^ RT group mice measured by ELISA (*n* = 5 per group). (**J**) Tumor growth curves of LLC subcutaneous tumors in corresponding groups (*n* = 7 to 8 per group). (**K**) Flow cytometry analysis of TCR Vγ4^+^ γδ T cell proportions of LLC subcutaneous tumors following radiotherapy (*n* = 5 per group). *P < 0.05; **P < 0.01; ***P < 0.001. Unpaired 2-tailed Student’s *t* test (**D**, **E**, **H**, **I**, and **K**), 1-way ANOVA with Tukey’s multiple comparisons test (**F** and **G**), 2-way ANOVA followed by Tukey’s multiple comparison test (**J**).

Given the well-established role of γδ T cells exerting immunomodulatory functions through secreting pleiotropic cytokines, we performed in-depth analysis of scRNA-seq data to characterize the phenotypic and functional features of γδ T cell populations in the postradiation TME. This revealed that γδ T cells were predominantly characterized by the expression of *Zbtb16*, *Il23r*, and *Il17a* (Figure 2A and Supplemental Figure 3A). Further subclustering analysis identified 7 transcriptionally distinct γδ T cell subsets, among which *Il17*^+^ γδ T cells (γδT17) emerged as the dominant subpopulation following radiotherapy (Figure 2, B and C, and Supplemental Figure 3B). These compelling results are consistent with previous studies demonstrating that *Zbtb16* and *Il23r* are essential regulators of γδ T cell differentiation and cytokine IL-17 production (14, 15). Meanwhile, we performed flow cytometry to quantify cytokine production in tumor-infiltrating γδ T cells, including IL-17, TNF-α, IFN-γ, IL-4, IL-10, and TGF-β. Among these cytokines, IL-17 exhibited the highest expression level in γδ T cells, and its production was further enhanced following radiotherapy (Figure 2, D and E, and Supplemental Figure 4, A–D). Both scRNA-seq and in vivo experimental data consistently demonstrated that IL-17–producing γδ T cells represent the predominant population in the TME after radiotherapy.

IL-17 is a prevalent proinflammatory cytokine that plays crucial roles in cancer progression and immune regulation (16). While CD4^+^ T helper cells (Th17) are traditionally considered the major source of IL-17 alongside γδT17 cells, we sought to identify the primary cellular source of IL-17 in the irradiated TME. We compared the infiltration dynamics of IL-17^+^ γδ T cells and IL-17^+^ αβ T cells at 3 distinct time points (24 hours, 96 hours, and 1 week) following 10 Gy or 2 Gy × 5 fractions irradiation. Notably, both cell populations reached their peak infiltration levels at 96 hours postirradiation, while IL-17^+^ γδ T cells substantially outnumbered IL-17^+^ αβ T cells at this time point (Figure 2, F–H, and Supplemental Figure 4, E–G). Notably, although the proportion of IL-17^+^ γδ T cells within the TME was reduced at 1 week postradiotherapy, immunological analysis of the TME at this time point revealed that TCRδ^–/–^ mice exhibited markedly elevated proportions of CD4^+^ T cells, CD8^+^ T cells, IFN-γ^+^ CD4^+^ T cells, and IFN-γ^+^ CD8^+^ T cells, alongside diminished proportions of Tregs and MDSCs, compared with WT mice (Supplemental Figure 4, H–N). This suggested that γδ T cells induced durable immunosuppressive and protumor effects following radiotherapy. Concurrently, the proportion of γ-H2AX^+^ cells in the tumor tissues was substantially decreased (Supplemental Figure 4O), indicating that most cells had completed the DNA damage repair process. This resolution of DNA damage may account for the observed reduction in γδ T cell frequency in the TME.

Cytokine profiling revealed that, while αβ T cells remained the primary source of IFNγ, γδ T cells constituted the dominant IL-17–producing population in the irradiated TME (Supplemental Figure 5A). ELISA analysis revealed a notable reduction (> 50%) in IL-17A levels in the tumor interstitial fluid of TCRδ^–/–^ mice compared with WT controls after radiotherapy (Figure 2I), further supporting the notion that γδ T cells were the dominant cell population responsible for IL-17 secretion in the TME following radiotherapy. To determine whether IL-17 secreted by γδ T cells mediates radiosensitization, we administered IL-17A–neutralizing antibodies to WT and TCRδ^–/–^ mice. IL-17A blockade enhanced radiosensitivity and prolonged postradiotherapy survival in WT mice, whereas such effect was absent in TCRδ^–/–^ mice (Figure 2J and Supplemental Figure 5B). Previous studies have reported that, in murine γδ T cells, Vγ4 and Vγ6 T cell subsets are predominantly associated with IL-17 production (17). Therefore, to further delineate the predominant γ chain subtypes of these γδ T cells, we performed flow cytometry and revealed that Vγ4^+^ γδ T cells constituted the predominant γδ T cell population in the irradiated TME (Figure 2K and Supplemental Figure 5C).

### γδ T cells attenuate radiosensitivity via MDSCs recruitment and T cell suppression.

**Figure 3:**
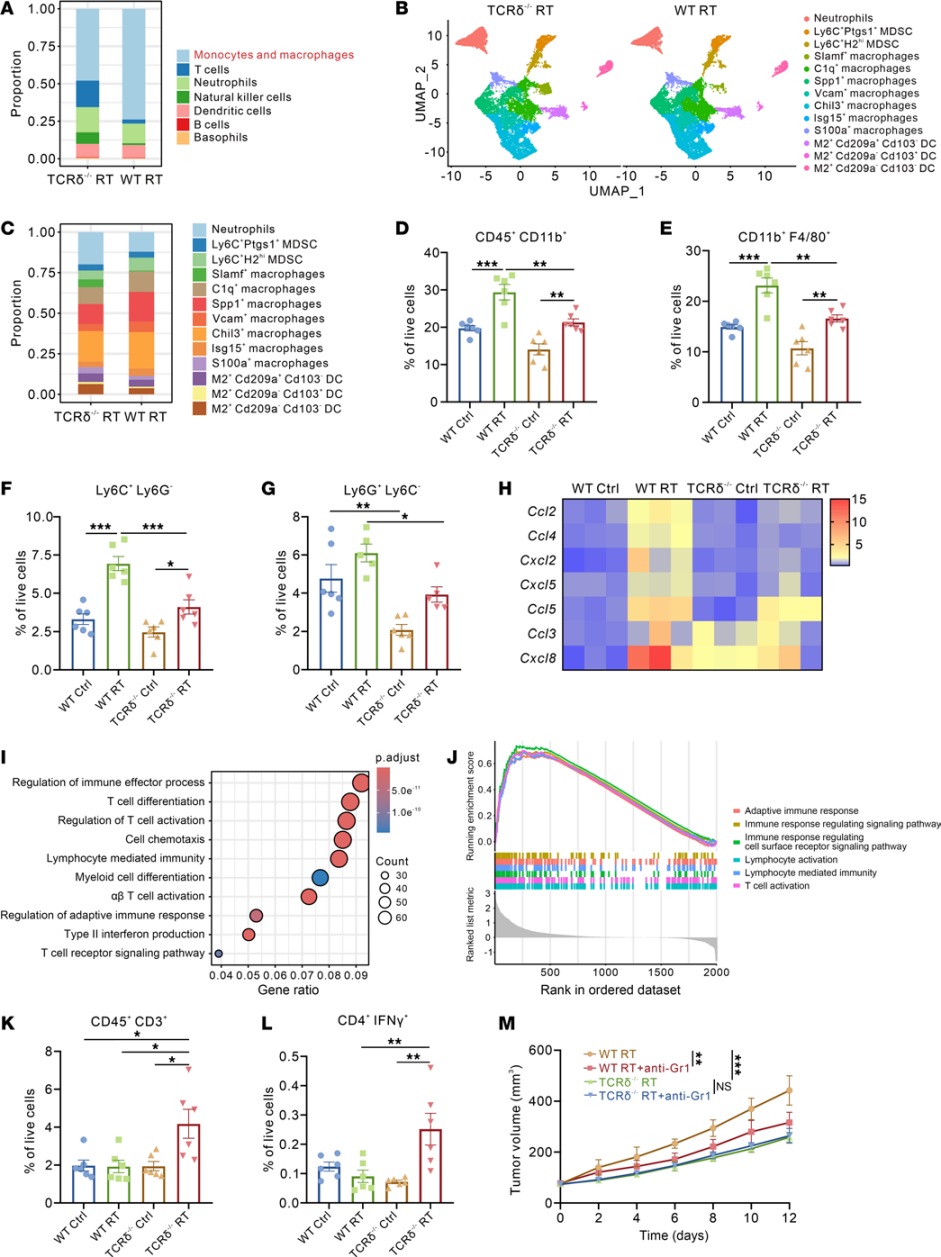
γδ T cell–mediated suppression of radiosensitivity through MDSC recruitment and T cell inhibition. (**A**) Stacked bar plot showing the proportion of major immune cell types originating from TCRδ^–/–^ RT and WT RT mice. (**B**) UMAP plot of monocytes and macrophages colored by cell clusters as indicated. (**C**) Stacked bar plot showing the proportion of major monocyte and macrophage clusters originating from TCRδ^–/–^ RT and WT RT mice. (**D**–**G**) Flow cytometry analysis of CD45^+^CD11b^+^ myeloid cell (**D**), CD11b^+^F4/80^+^ macrophage (**E**), Ly6C^+^Ly6G^–^ M-MDSC (**F**), and Ly6G^+^Ly6C^–^ PMN-MDSC (**G**) proportions in the TME of LLC subcutaneous tumors in corresponding groups (*n* = 5–6 per group). (**H**) Heatmap of MDSC-related chemokine expression from LLC subcutaneous tumors in corresponding groups. Data presented as the mean of 3 biological replicates. (**I**) GO enrichment analysis of differentially expressed genes in LLC subcutaneous tumors from TCRδ^–/–^ RT and WT RT mice. (**J**) GSEA enrichment analysis of differentially expressed genes in LLC subcutaneous tumors from TCRδ^–/–^ RT and WT RT mice. (**K** and **L**) Flow cytometry analysis of CD45^+^CD3^+^ T cell (**K**) and CD4^+^IFNγ^+^ Th1 cell (**L**) proportions in the TME of LLC subcutaneous tumors in corresponding groups (*n* = 6 per group). (**M**) Tumor growth curves of LLC subcutaneous tumors in corresponding groups (*n* = 7 per group). *P < 0.05; **P < 0.01; ***P < 0.001. 1-way ANOVA with Tukey’s multiple comparisons test (**D**–**G**, **K**, and **L**), 2-way ANOVA followed by Tukey’s multiple comparison test (**M**).

To elucidate the potential mechanisms underlying γδ T cell–mediated radioresistance, we performed scRNA-seq analysis on CD45^+^ immune cells from Lewis subcutaneous tumors in WT mice and TCRδ^–/–^ mice after 10 Gy irradiation (TCRδ^–/–^ RT vs WT RT). TCRδ^–/–^ mice exhibited reduced monocyte and macrophage enrichment compared with WT mice (Figure 3A). Thus, we further distinguished myeloid-derived cells into 13 distinct subtypes based on differential gene expression (Supplemental Figure 6A). Compared with TCRδ^–/–^ mice, WT mice showed increased macrophages and MDSC infiltration but reduced neutrophils and dendritic cells (DCs) accumulation post-irradiation (Figure 3, B and C). Flow cytometric analysis confirmed increased proportions of myeloid cell populations, including macrophages (CD11b^+^ F4/80^+^), M-MDSCs (Ly6C^+^ Ly6G^–^), and PMN-MDSCs (Ly6G^+^ Ly6C^–^) in WT mice versus TCRδ^–/–^ mice (Figure 3, D–G, and Supplemental Figure 6B). Based on the recognized role of MDSCs in suppressing antitumor immunity (18), we speculated that elevated γδ T cells after radiation mediate radioresistance through facilitating the recruitment of MDSCs within the TME. RT-qPCR analysis of subcutaneous tumors revealed marked upregulation of multiple MDSC-associated chemokines, including CCL2 and CCL3, in irradiated WT mice compared with TCRδ^–/–^ mice (Figure 3H), suggesting that γδ T cells may exert their functional effects through MDSC recruitment.

Furthermore, Gene Ontology (GO) enrichment analysis of differentially expressed genes between irradiated subcutaneous tumors from TCRδ^–/–^ and WT mice revealed enrichment of T cell–related immune response and immune activation pathways, such as “Regulation of immune effector process”, “T cell differentiation”, “Lymphocyte-mediated immunity”, and “α β T cell activation” (Figure 3I). Consistently, GSEA demonstrated the upregulation of immunoregulatory pathways in TCRδ^–/–^ subcutaneous tumors, including “Adaptive immune response”, “Immune response regulating signaling pathway”, “Lymphocyte mediated immunity” and “T cell activation” (Figure 3J). In subcutaneous tumor models of WT and TCRδ^–/–^ mice, we quantified tumor-infiltrating T cell populations using flow cytometry and found that, compared with WT mice, TCRδ^–/–^ mice exhibited substantially increased T cell infiltration, particularly CD3^+^CD4^+^IFNγ^+^ Th1 cells, in the TME following radiotherapy (Figure 3, K and L, and Supplemental Figure 6C). In contrast, CD3^+^CD8^+^GrzB^+^ T cell and CD4^+^FoxP3^+^ Treg proportions remained comparable between 2 groups (Supplemental Figure 6, D and E). Finally, to determine whether γδ T cell–mediated radioresistance is MDSC dependent, we performed in vivo MDSCs depletion experiments via Gr-1 antibody in tumor-bearing WT and TCRδ^–/–^ mice. The clearance efficiency of the Gr-1 antibody in spleen and peripheral blood was more than 95% (Supplemental Figure 7, A and B), and tumor growth curves revealed that MDSC depletion enhanced radiosensitivity and prolonged survival in WT mice, while no such effect was observed in TCRδ^–/–^ mice (Figure 3M and Supplemental Figure 7C). Collectively, these results suggested that γδ T cells promote radioresistance by recruiting MDSCs, which subsequently suppress the T cell–mediated antitumor immune responses.

### Radiation-induced macrophage-derived CCL20 facilitates γδ T cell recruitment.

**Figure 4:**
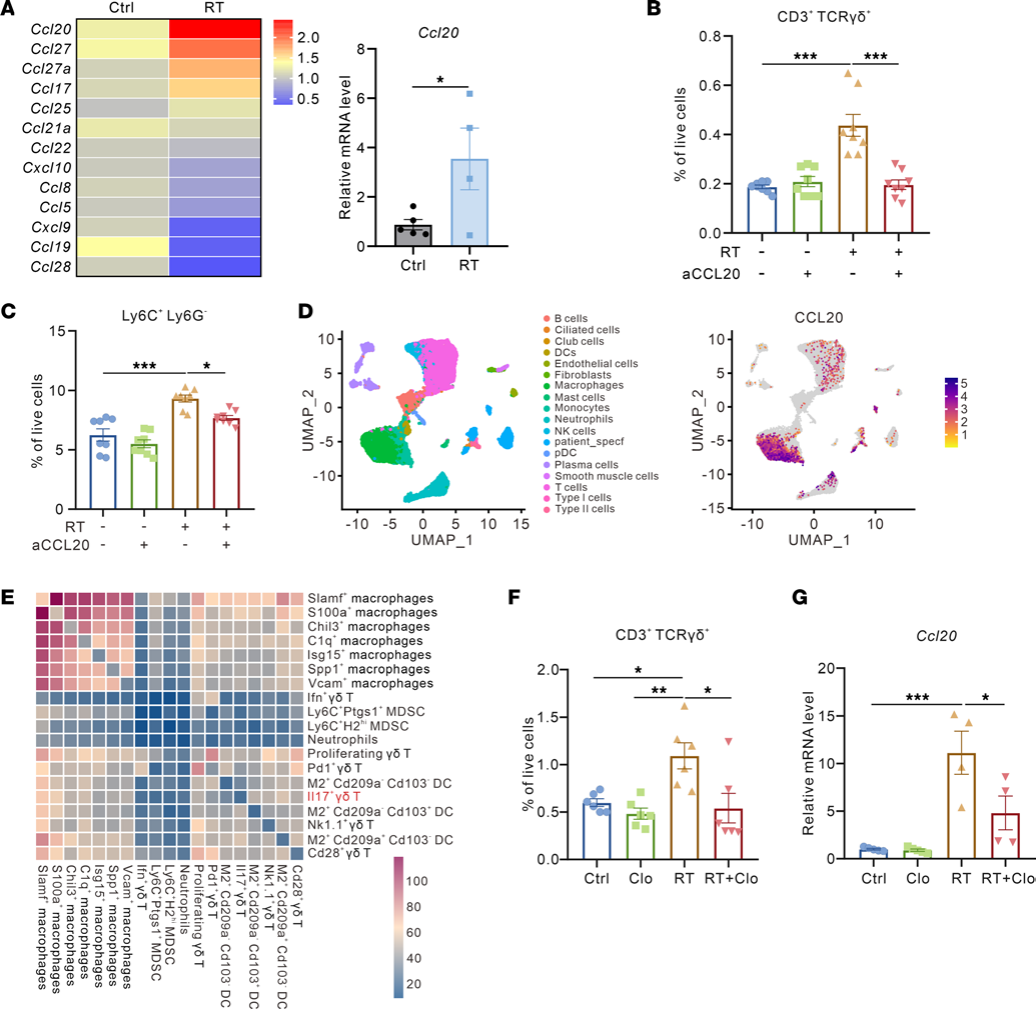
Macrophage-derived chemokine CCL20 promotes γδ T cell recruitment. (**A**) Left panel, heatmap of T cell–related chemokines expression from LLC subcutaneous tumors after radiotherapy. Right panel, relative mRNA expression of *Ccl20* from LLC subcutaneous tumors in corresponding groups (*n* = 4–6 per group). (**B** and **C**) Flow cytometry analysis of γδ T cell (**B**) and Ly6C^+^Ly6G^–^ M-MDSC (**C**) proportions in the TME of LLC subcutaneous tumors in corresponding groups (*n* = 8 per group). (**D**) Left panel, UMAP plot of major cell clusters from human lung cancer samples (GSE127465) colored by cell identities. Right panel, feature plots of the *CCL20* expression in the major cell clusters. (**E**) Correlation between monocyte and macrophage types (estimated from matched scRNA-seq) and γδ T cell clusters. (**F**) Flow cytometry analysis of γδ T cell proportions in the TME of LLC subcutaneous tumors after macrophage clearance (*n* = 6 per group). (**G**) Relative mRNA expression of *Ccl20* from LLC subcutaneous tumors after macrophage clearance (*n* = 4–5 per group). *P < 0.05; **P < 0.01; ***P < 0.001. Unpaired 2-tailed Student’s *t* test (**A**), 1-way ANOVA with Tukey’s multiple comparisons test (**B**, **C**, **F**, and **G**).

To elucidate mechanisms responsible for radiation-induced γδ T cell infiltration, we quantified the expression of multiple T cell–related chemokines in Lewis subcutaneous tumors following 10 Gy irradiation. RT-qPCR analysis identified *Ccl20* as the most pronouncedly upregulated chemokine after radiation (Figure 4A). It has been reported that CCL20-CCR6 axis was essential for IL-17A–producing γδ T cell recruitment (19). Therefore, we utilized CCL20 neutralizing antibody combined with radiotherapy in WT mice and found CCL20 blockade effectively reversed radiation-induced γδ T cell accumulation in the TME (Figure 4B), establishing CCL20 as a critical driver of γδ T cell recruitment in irradiated tumors. Meanwhile, CCL20 neutralization also attenuated M-MDSC and PMN-MDSC accumulation after radiation (Figure 4C and Supplemental Figure 8A).

Given the critical role of CCL20 in mediating γδ T cell recruitment, we next sought to determine its major cellular origin after radiation. Integrated analysis of scRNA-seq data from human lung cancer tissues revealed that macrophages displayed marked enrichment of *CCL20* transcripts (20), exhibiting higher expression levels compared with other cell subsets (Figure 4D and Supplemental Figure 8B). Cell-cell interaction analysis based on our previous scRNA-seq data demonstrated that macrophages represent the predominant interacting populations with γδ T cells in irradiated WT mice, consistent with their role as primary CCL20 producers (Figure 4E). Additionally, we employed clodronate liposomes (Clo) in vivo to systemically deplete macrophages; flow cytometry analysis demonstrated that Clo-mediated macrophage ablation markedly attenuated radiation-induced γδ T cell accumulation in the TME (Figure 4F and Supplemental Figure 8, C and D). Concomitantly, RT-PCR analysis revealed that macrophage depletion markedly reduced *Ccl20* expression levels in irradiated subcutaneous tumors (Figure 4G). Taken together, the aforementioned results suggested that macrophage-secreted chemokine CCL20 in the irradiated TIME plays a pivotal role in mediating γδ T cell recruitment.

### Radiated tumor cell–released RT-MPs leads to Ccl20 upregulation in macrophages via cGAS-STING/NF-κB signaling pathway.

**Figure 5:**
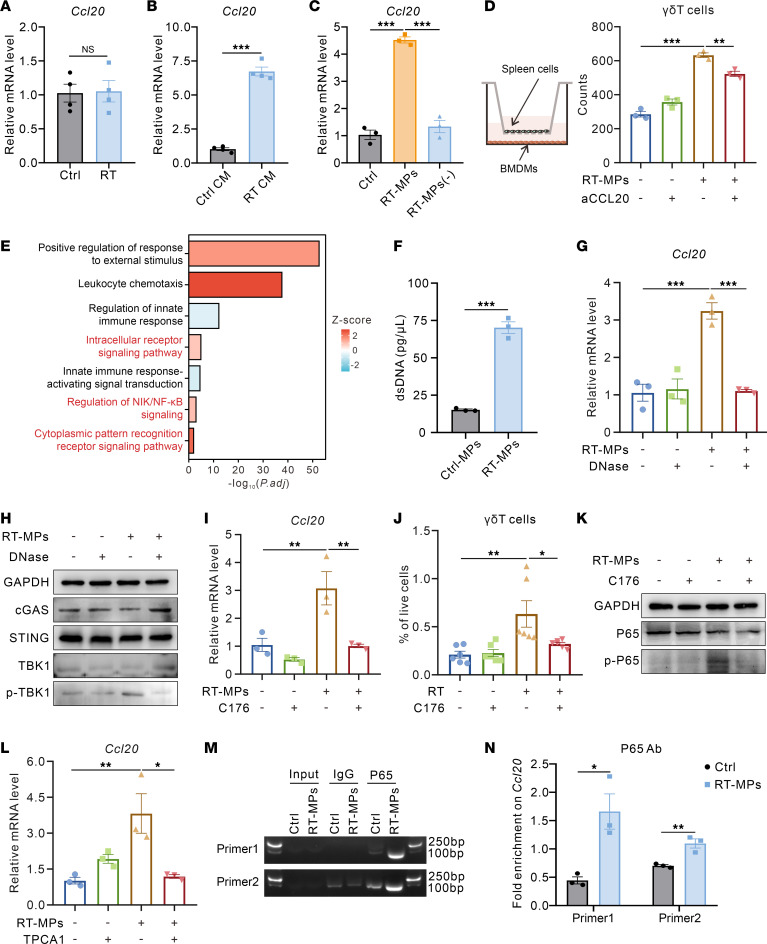
RT-MPs upregulate *Ccl20* expression in macrophages through cGAS-STING/NF-κB pathway activation. (**A**) Relative mRNA expression of *Ccl20* in BMDMs after 10 Gy irradiation for 24 hours. (**B**) Relative mRNA expression of *Ccl20* in BMDMs treated with conditioned medium (CM) from control or irradiated tumor cells. (**C**) Relative mRNA expression of *Ccl20* in BMDMs treated with RT-MPs or irradiated tumor cell–derived CM depleted of RT-MPs. (**D**) Left panel, pattern diagram of transwell migration assay. Right panel, flow cytometry analysis of γδ T cell migration to the lower chamber in corresponding treatment conditions. (**E**) GO enrichment analysis of differentially expressed genes in LLC subcutaneous tumors from WT RT and WT Ctrl mice. (**F**) Quantitative measurement of dsDNA content in RT-MPs and Ctrl-MPs. (**G**) Relative mRNA expression of *Ccl20* in BMDMs treated with RT-MPs or dsDNA-depleted RT-MPs. (**H**) Representative Western blot images showing protein expression levels of cGAS, STING, TBK1, and p-TBK1 in BMDMs. (**I**) Relative mRNA expression of *Ccl20* in BMDMs treated with RT-MPs or the STING inhibitor C176. (**J**) Flow cytometry analysis of γδ T cell proportions in the TME of LLC subcutaneous tumors in corresponding groups (*n* = 6 per group). (**K**) Representative Western blot images showing protein expression levels of P65 and p-P65 in BMDMs. (**L**) Relative mRNA expression of *Ccl20* in BMDMs treated with RT-MPs or the NF-κB pathway inhibitor TPCA1. (**M** and **N**) ChIP assay of P65 in RT-MP–treated BMDMs. Representative gel electrophoresis results are shown in **M**. P65 binding to the *Ccl20* promoter region is quantified by qPCR, with results expressed as fold enrichment in site-specific occupancy relative to the control (**N**). *P < 0.05; **P < 0.01; ***P < 0.001. Unpaired 2-tailed Student’s *t* test (**A**, **B**, **F**, and **N**), 1-way ANOVA with Tukey’s multiple comparisons test (**C**, **D**, **G**, **I**, **J**, and **L**).

To unravel the potential mechanisms driving radiation-induced macrophage CCL20 upregulation, we investigated whether radiation directly enhances *Ccl20* expression in macrophages. RT-qPCR analysis revealed that direct 10 Gy irradiation failed to upregulate *Ccl20* expression in bone marrow–derived macrophages (BMDMs) in vitro (Figure 5A), suggesting that microenvironmental factors or cell-cell interactions may indirectly regulate macrophage gene expression. Given that tumor cells represent the predominant cellular component of the TME, we hypothesized that radiation-induced tumor cell–derived factors might mediate this indirect regulation. Using conditioned medium (CM) from 10 Gy–irradiated tumor cells, we observed considerable upregulation of *Ccl20* expression in macrophages compared with control CM (Figure 5B). To further identify the specific components in the supernatant of irradiated tumor cells that mediate this effect, we focused on extracellular vesicles (EVs) based on their crucial roles in mediating intercellular communication (21). Since our previous studies have demonstrated and characterized that irradiated tumor cell–derived microparticles (RT-MPs) exhibit potent tumoricidal and immunostimulatory properties (22), we wondered whether RT-MPs mediate CCL20 secretion by macrophages, thereby promoting γδ T cell infiltration. We isolated RT-MPs from irradiated tumor cell supernatants and found that RT-MPs upregulated *Ccl20* expression in BMDMs, whereas RT-MP-depleted conditioned medium lost this capacity (Figure 5C and Supplemental Figure 9A). Subsequently, we employed the transwell chemotaxis assays in vitro, with spleen single cells seeded in the upper chamber of a 3 μm transwell insert and BMDMs placed in the lower chamber. After 24 hours of coculture, we observed that RT-MPs markedly enhanced the capacity of macrophages to recruit γδ T cells, but the effect was attenuated by CCL20 neutralization (Figure 5D). Furthermore, direct intratumoral injection of RT-MPs pronouncedly enhanced γδ T cell infiltration within the TME (Supplemental Figure 9B).

Mechanistically, GO enrichment analysis of differentially expressed genes between irradiated and control subcutaneous tumors in WT mice revealed enrichment of “intracellular receptor signaling pathway”, “regulation of NIK/NF-κB signaling”, and “cytoplasmic pattern recognition receptor (PRR) signaling pathway” (Figure 5E). It has been well established that irradiation-induced DNA double-strand breaks (DSBs) activate PRRs, such as cyclic GMP-AMP synthase (cGAS), through cytoplasmic DNA fragment release, thereby regulating antitumor immunity (23). Therefore, we quantified the dsDNA levels and observed roughly a 3-fold increase in dsDNA content in RT-MPs compared with microparticles from nonirradiated tumor cells (Figure 5F and Supplemental Figure 9C). Taking these findings into account, we hypothesized that dsDNA encapsulated within RT-MPs activates the cGAS-STING pathway in macrophages, promoting CCL20 upregulation. DNase I–mediated depletion of dsDNA in RT-MPs markedly attenuated their capacity to upregulate *Ccl20* expression in macrophages (Figure 5G and Supplemental Figure 9, D and E). Consistent with this finding, Western blot analysis demonstrated that RT-MPs robustly activated the cGAS-STING signaling pathway in macrophages, while DNase I pretreatment completely reversed this activation (Figure 5H and Supplemental Figure 9F). Moreover, STING inhibitor C176 or genetic knockdown of STING via siRNA transfection in macrophages substantially attenuated RT-MP–induced *Ccl20* upregulation (Figure 5I and Supplemental Figure 9, G–I). In vivo administration of C176 combined with radiotherapy reduced the proportions of tumor-infiltrating γδ T cells (Figure 5J). However, C176 did not markedly alter the proportions of M-MDSCs and PMN-MDSCs after radiotherapy, which may be related to the prominent role of cGAS-STING pathway in radiation-induced adaptive immune activation (3) (Supplemental Figure 9, J and K).

Activation of the cGAS-STING pathway through cytosolic DNA sensing has been shown to trigger downstream NF-κB signaling, thereby amplifying inflammatory responses (24). Therefore, we hypothesized that RT-MPs might regulate macrophage *Ccl20* expression through NF-κB activation downstream of cGAS-STING. Western blot analysis confirmed that RT-MPs activated the NF-κB pathway in macrophages, as evidenced by increased phosphorylation of P65 at Ser468. Both STING inhibitor C176 and genetic STING knockdown markedly attenuated RT-MP–induced P65 phosphorylation (Figure 5K and Supplemental Figure 9, L and M). Meanwhile, either NF-κB inhibitor TPCA-1 or siRNA-mediated P65 knockdown reversed RT-MP–driven *Ccl20* upregulation in macrophages (Figure 5L and Supplemental Figure 9, N–P). Upon activation of the NF-κB pathway, the P65 subunit typically translocates to the nucleus, where it functions as the transcription factor to regulate gene expression (25). P65 chromatin immunoprecipitation (ChIP) sequencing data of macrophages in the ENCODE project suggested a potential P65 binding sites around the promotor region of *Ccl20* (Supplemental Figure 9Q). ChIP assay of P65 followed by DNA gel electrophoresis and quantitative PCR identified that RT-MP treatment markedly enhanced the P65 binding to the promoter regions of *Ccl20* in BMDMs (Figure 5, M and N), directly linking NF-κB activation to *Ccl20* transcriptional regulation. In a word, these results suggested that RT-MP–encapsulated dsDNA triggers cGAS-STING/NF-κB signaling axis, leading to transcriptional activation of *Ccl20* in macrophages.

### Radiation-induced γδ T cell infiltration impairs the efficacy of radiotherapy combined with immunotherapy.

**Figure 6:**
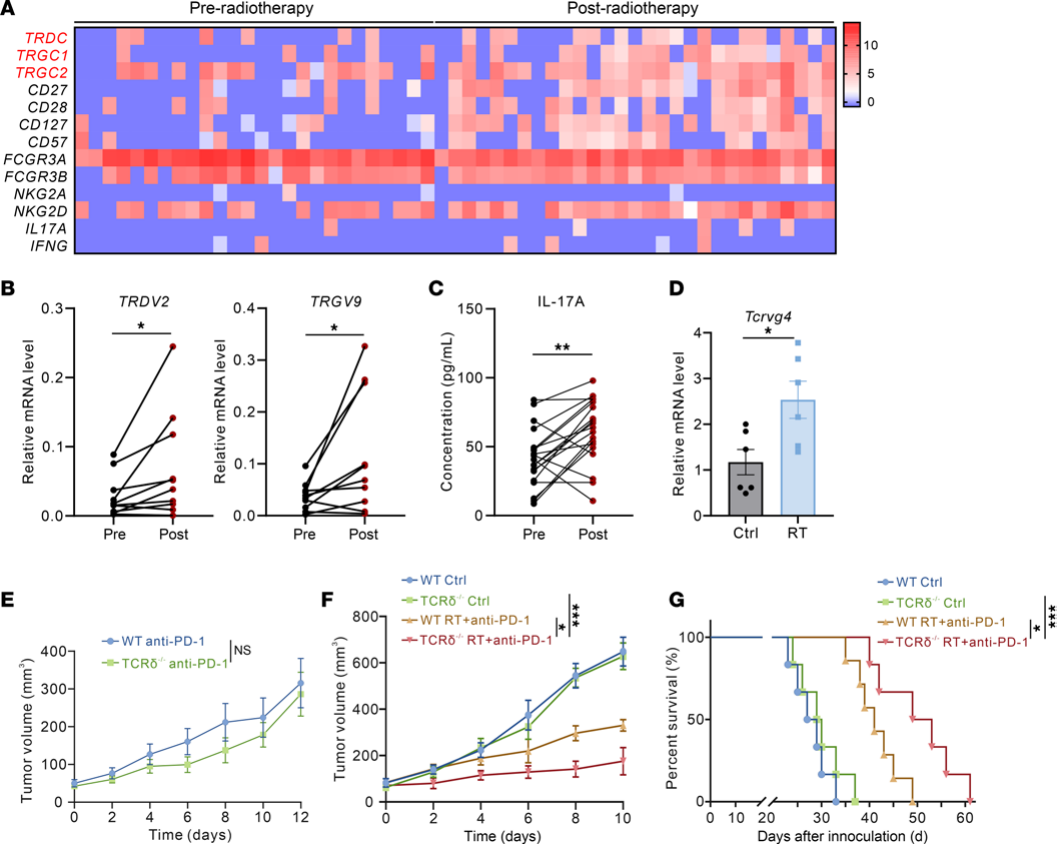
Clinical relevance between γδ T cells and radiotherapy. (**A**) Transcriptomic analysis of pancreatic cancer patient samples shows increased expression of TCR-encoding genes in γδ T cells after radiotherapy (GSE225767). (**B**) Relative mRNA expression of *TRDV2* and *TRGV9* in peripheral blood PBMCs from patients with lung cancer before and after radiotherapy (*n* = 10 paired samples). (**C**) IL-17A concentrations in plasma from lung cancer patients before and after radiotherapy measured by ELISA (*n* = 19 paired samples). (**D**) Relative mRNA expression of *Tcrvg4* in peripheral blood PBMCs from LLC subcutaneous tumor-bearing mice after radiotherapy (*n* = 6 per group). (**E**) Tumor growth curves of LLC subcutaneous tumors in corresponding groups with anti-PD-1 treatment (*n* = 6–9 per group). (**F**) Tumor growth curves of LLC subcutaneous tumors in corresponding groups (*n* = 6–7 per group). (**G**) Kaplan-Meier survival plot of LLC lung cancer-bearing mice in the corresponding groups (*n* = 6–7 per group). *P < 0.05; **P < 0.01; ***P < 0.001. Paired 2-tailed *t* test (**B** and **C**), Unpaired 2-tailed Student’s *t* test (**D**), 2-way ANOVA (**E** and **F**), Log-rank (Mantel-Cox) test (**G**).

To validate our findings in clinical specimens, we analyzed the publicly available transcriptomic sequencing data from Piper et al., comprising pre- and postneoadjuvant radiotherapy tissues from patients with pancreatic ductal adenocarcinoma (PDAC) (26). The analysis revealed marked upregulation of γδ T cell–related gene expression profiles in postradiotherapy tumor tissues (Figure 6A). In addition, we collected paired peripheral blood samples from patients with non-small cell lung cancer (NSCLC) before and after radiotherapy. Analysis of peripheral blood mononuclear cells (PBMCs) by RT-qPCR demonstrated considerable upregulation of γδ T cell–specific genes (*TRDV2* and *TRGV9*) after radiotherapy (Figure 6B). Immunofluorescence staining confirmed increased proportions of IL-17A^+^ γδ T cells in postradiotherapy PBMCs (Supplemental Figure 10A). Consistent with these findings, ELISA measurements showed markedly elevated serum IL-17A levels in patients after radiotherapy (Figure 6C). Similarly, we detected notable upregulation of γδ T cell–related gene (*Tcrvg4*) in PBMCs of irradiated mice (Figure 6D). These coordinated changes collectively support radiation-induced γδT17 cell infiltration.

Based on our previous findings that γδ T cells exhibit immunosuppressive properties and mediate radioresistance, we further investigated whether γδ T cell deletion could potentiate the therapeutic efficacy of immune checkpoint blockade (ICB) monotherapy or its combination with radiotherapy. While anti–PD-1 monotherapy demonstrated similar antitumor effects in WT and TCRδ^–/–^ mice (Figure 6E), flow cytometry analysis of the TME showed comparable infiltration levels of total γδ T cells, IL-17–producing γδ T cells, and IFN-γ–producing γδ T cells between anti-PD-1–treated and untreated controls (Supplemental Figure 10B). Strikingly, in the combination therapy group receiving both radiotherapy and anti–PD-1 treatment, TCRδ^–/–^ mice exhibited markedly slower tumor growth and prolonged survival compared with WT mice (Figure 6, F and G), suggesting that γδ T cell ablation enhances the therapeutic efficacy of combined radioimmunotherapy.

## Discussion

In this study, we systematically elucidated the characteristics and functional mechanisms of γδ T cells in the TME after radiotherapy, revealing their role in the promotion of radioresistance. Mechanistically, it was demonstrated that radiotherapy triggers the release of dsDNA-containing RT-MPs from tumor cells, which activate the cGAS-STING/NF-κB signaling pathway in macrophages, leading to the upregulation of CCL20 expression. The chemokine CCL20 recruits γδ T cells, which serve as the primary source of IL-17 in the postradiotherapy TME. These γδ T cells facilitate radioresistance by promoting the infiltration of MDSCs and suppressing T cell–mediated antitumor immunity. Our findings provide valuable insights into the mechanisms underlying radioresistance and highlight potential therapeutic targets for enhancing radiotherapy efficacy.

γδ T cells represent a heterogeneous subset of T lymphocytes characterized by the expression of γδ T cell receptors. In humans, they are classified into at least 3 major subsets based on the TCRδ chain: Vδ1, Vδ2, and Vδ3 T cells (11). Among these, Vδ2 T cells dominate the peripheral circulation, constituting 60%–95% of the γδ T cell population, and predominantly pair with the Vγ9 chain to form Vγ9Vδ2 T cells (27, 28). In mice, γδ T cells are categorized based on the TCR-γ chain into Vγ1, Vγ4, Vγ5, Vγ6, and Vγ7 subsets (29). Recent studies have highlighted the remarkable heterogeneity and plasticity of γδ T cells, revealing their dual roles in antitumor immunity. On one hand, certain subsets, such as human Vγ9Vδ2 T cells and murine Vγ1 and Vγ4 T cells, exert antitumor effects by secreting cytokines like IFN-γ and TNF-α and directly mediating tumor cell cytotoxicity (30). On the other hand, other subsets, including human Vδ1 T cells and murine Vγ4 and Vγ6 T cells, promote tumor angiogenesis and immune suppression through the secretion of IL-17 and amphiregulin (AREG), thereby facilitating tumor immune escape (31, 32). However, the specific gene expression profiles and functional roles of γδ T cells in the context of radiotherapy remain incompletely elucidated. In this study, we demonstrated that γδ T cells in the TME after radiotherapy promote radioresistance. ScRNA-seq revealed that these cells are characterized by elevated expression of *Zbtb16*, *Il23r*, and *Il17a*. Previous studies have reported that *Zbtb16* and *Il23r* are critical for γδ T cell differentiation and IL-17A production (33, 34), which aligns with our conclusions. Our study addresses a critical gap in understanding the role of γδ T cells in radiotherapy sensitivity and suggests that γδ T cells are critical therapeutic targets.

Notably, with the widespread clinical adoption of immune checkpoint inhibitors targeting PD-1/PD-L1, radioimmunotherapy combinations have emerged as a cornerstone treatment for multiple malignancies. The landmark PACIFIC trial, establishing consolidation durvalumab after chemoradiation for locally advanced NSCLC, exemplifies the therapeutic promise of this approach (35, 36). However, radiotherapy exerts dualistic immunomodulatory effects: while it activates cGAS-STING signaling through radiation-induced DNA damage and ROS generation, thereby promoting type I IFN–mediated antitumor immunity (3, 37), it concurrently recruits and activates immunosuppressive populations including Tregs, tumor-associated neutrophils (TANs), tumor-associated macrophages (TAMs), and MDSCs in the TME (38–41). Our study revealed that γδ T cell ablation substantially enhanced the efficacy of radioimmunotherapy. Mechanistically, we demonstrated that radiation-recruited γδ T cells facilitate MDSCs accumulation and suppress T-cell activation, thereby promoting tumor progression. These findings deepen our understanding of radioimmunobiology and provide actionable insights for optimizing clinical radioimmunotherapy regimens.

The IL-17 family comprises six members, IL-17A to IL-17F, with IL-17A being the best characterized and most prominent cytokine in this family (42). Unless otherwise specified, IL-17 typically refers to IL-17A. Although Th17 cells are often regarded as the primary source of IL-17, emerging evidence indicates that other immune cells, including NKT cells, CD8^+^ T cells, γδ T cells, dendritic cells, and macrophages, also produce IL-17 (43). IL-17 is a pleiotropic proinflammatory cytokine essential for host immune defense, tissue repair, inflammatory disease pathogenesis, and cancer progression (44). Aberrant IL-17 levels have been implicated in the development and progression of various malignancies, including breast, liver, pancreatic, and lung cancers (45–47). IL-17–induced chronic inflammation is also recognized as a critical factor mediating cellular transformation, promoting tumor cell proliferation and metastasis, and inducing immune tolerance (48). However, some studies have revealed unique antitumor roles of the IL-17 family. For example, Timothy et al. reported that IL-17D mediates tumor rejection by recruiting NK cells, thereby suppressing tumor progression (49). In fact, the dual roles of IL-17 in promoting or inhibiting tumor growth may be context dependent, with its production levels and duration likely determining its effects (48). Transient IL-17 activity typically activates inflammatory signaling pathways, potentially inducing acute inflammation to eliminate pathogens. In contrast, sustained or excessive IL-17 may promote tumorigenic processes. In this study, we identified γδ T cells, rather than Th17 cells, as the primary source of IL-17 in the TME after radiotherapy and demonstrated their critical role in driving radioresistance and tumor progression. Therefore, combining IL-17 signaling blockade with radiotherapy may achieve better tumor suppression efficacy. Furthermore, future research could explore whether IL-17 levels can serve as a predictive biomarker for radiotherapy sensitivity, paving the way for more personalized treatment strategies.

Extensive studies have established that the local TME plays a critical role in reshaping the activation and differentiation of γδ T cells (50). An immunosuppressive TME can impede the antitumor efficacy of γδ T cells and promote their polarization toward an immunosuppressive phenotype. The TME orchestrates complex crosstalk between γδ T cells and various immune cell populations, including αβ T cells, B cells, dendritic cells, macrophages, monocytes, natural killer cells, and neutrophils (51). Notably, macrophages have been shown to recruit Vγ9Vδ2 T cells to the site of infection through CXCL10 and CXCR3 receptor-ligand interactions. Subsequently, Vδ2^+^ γδ T cells elicit localized cytotoxic responses by releasing perforin and granzymes (52). Conversely, IFN-γ and TNF-α secreted by activated Vγ9Vδ2 T cells can induce cyclooxygenase-2 (COX2) expression and prostaglandin E2 release in macrophages, which, in turn, downregulate the cytotoxic activity of γδ T cells and facilitate tumor immune evasion (52, 53). In this study, we provide compelling evidence that the chemokine CCL20, secreted by macrophages following radiotherapy, plays a pivotal role in recruiting γδ T cells, further advancing our understanding of the intricate interplay among diverse immune cell populations within the TME.

Extracellular vesicles serve as critical mediators of intercellular communication. Our team pioneered the discovery that RT-MPs mediate radiation-induced bystander effects, exerting potent antitumor efficacy by inducing ferroptosis and remodeling the TME (22, 54). Additionally, RT-MPs markedly upregulate the expression of MHC-I molecules on nonirradiated tumor cells, thereby promoting T cell–mediated recognition and cytotoxicity (55). However, the precise components within RT-MPs responsible for these effects remain poorly understood. In this study, we have revealed that dsDNA encapsulated within RT-MPs activates the cGAS-STING signaling pathway in macrophages, leading to increased expression of the chemokine CCL20. This effect was reversed upon DNase-mediated digestion of dsDNA within RT-MPs. These findings not only deepen our mechanistic understanding of RT-MP–mediated intercellular communication, but also provide distinct insights into the role of EVs in mediating intercellular material transfer and immune regulation.

Immunotherapeutic strategies based on γδ T cells have garnered increased attention due to their MHC-independent antigen recognition and robust antitumor activity. Current clinical trials have demonstrated remarkable potential for adoptive cell therapy using Vγ9Vδ2 T cells and bispecific antibodies (56). For instance, the adoptive transfer of expanded Vγ9Vδ2 T cells in patients with advanced hepatocellular carcinoma and lung cancer has substantially improved overall survival, demonstrating favorable safety and efficacy (30). However, the clinical application of these strategies is complicated, largely due to the complexity of the TME and the dualistic functionality of γδ T cells (50). In this study, we observed that γδ T cells exhibited immunosuppressive properties and mediated resistance to radiotherapy in the murine model. Furthermore, analysis of peripheral blood samples from clinical patients before and after radiotherapy revealed an increased proportion of γδ T cells after radiotherapy, accompanied by a marked elevation in IL-17 levels. Despite these insights, several limitations must be acknowledged. First, there are notable differences in the distribution, phenotype, and functionality of γδ T cells between humans and mice, necessitating caution when translating murine findings to clinical settings. Second, the precise targeting of specific γδ T cell subsets in clinical practice remains a formidable technical hurdle. Our findings suggest that exploring the upstream and downstream mechanisms governing γδ T cell functions in the TME after radiotherapy may offer alternative therapeutic avenues. Specifically, targeting the IL-17 signaling pathway or the chemokine CCL20 may potentially enhance radiosensitivity. As research on γδ T cells continues to advance, our study provides valuable insights and a foundation for developing γδ T cell–based radiosensitization strategies.

Undoubtedly, the tumor microenvironment orchestrates radiation responses through intrinsically complex regulatory networks. Diverse cellular subsets and cytokine/chemokine cascades interact dynamically to form an interconnected signaling web (57, 58). Beyond the immunosuppressive γδ T cell/MDSC axis identified in our study, alternative mechanisms undoubtedly contribute to radioresistance. For instance, TGF-β secreted by irradiated cancer-associated fibroblasts potently drives the acquisition of radioresistant properties in cancer stem cells (59). These limitations highlight the need to explore additional cellular players and signaling pathways in future work, particularly focusing on the spatiotemporal dynamics of microenvironmental reprogramming after radiotherapy.

In summary, our findings elucidate the role and underlying mechanisms by which γδ T cells mediate radioresistance. Irradiated tumor cells release dsDNA-containing microparticles, which activate the cGAS-STING/NF-κB pathway in macrophages and upregulate chemokine CCL20 to recruit γδ T cells. Within the TME, γδ T cells characterized by the expression of *Zbtb16*, *Il23r*, and *Il17a* serve as the primary source of IL-17, fostering an immunosuppressive milieu and driving radioresistance. These findings provide a strong rationale for developing γδ T cell–targeted strategies to enhance radiosensitivity, offering a promising approach to overcoming therapeutic resistance in cancer.

## Methods

### Sex as a biological variable.

Our mouse study examined male and female animals, and similar findings are reported for both sexes. In our peripheral blood samples before and after radiotherapy, both male and female patients are included. The biological variable observed in the experiment was the effect of radiotherapy on γδ T cells, and sex was not considered as an observation variable.

### Cell lines and cell culture.

The murine Lewis lung carcinoma (LLC) cell line and murine melanoma cell line B16-F10 were supplied by the American Tissue Culture Collection (ATCC). LLC cells were cultured in Dulbecco’s Modified Eagle Medium (DMEM), whereas B16-F10 cells were maintained in RPMI-1640 medium. Both media were supplemented with 10% fetal bovine serum (FBS) and 1% penicillin/streptomycin. All cells were incubated at 37°C with 5% CO_2_.

### Mice.

Female and male C57BL/6J mice (6 weeks old) were purchased from Wuhan Moubaili Biotechnology Co. Ltd. TCRδ^–/–^ mice were provided by Zhinan Yin at Jinan University (Guangzhou, China). All animal care and experimental procedures were conducted in accordance with the guidelines of the Animal Experimentation Ethics Committee of Huazhong University of Science and Technology (HUST, Wuhan, China).

### Chemical reagents.

STING inhibitor C-176 (Selleck, S6575), NF-κB inhibitor TPCA-1 (MCE, HY-10074), Mouse IL-17A neutralization antibody (BioXCell, BE0173), and Mouse CCL20 neutralization antibody (R&D Systems, AF760-SP) were administered according to the indicated protocols.

### Radiation.

Irradiation was performed using Varian Trilogy linear accelerator with a 6-MV X-ray beam quality and 600 cGy/min dose rate. Radiation doses were verified using thermos-luminescent dosimeters (TLDs). LLC cells and BMDMs were exposed to a single dose of 10 Gy. For the subcutaneous tumor, mice were anesthetized and radiation was delivered to the right posterior limbs (with tumors) using either single-dose (10 Gy or 8 Gy) or fractionated (2 Gy × 5) protocols.

### scRNA-seq.

Single-cell suspensions were prepared from subcutaneous LLC tumors of WT and TCRδ^–/–^ mice at 96 hours after 10-Gy irradiation. To isolate CD45^+^ leukocytes for scRNA-seq, the suspensions were first incubated with purified anti-mouse CD16/32 antibody (Biolegend, 101302) and Zombie NIR Fixable Viability Kit (Biolegend, 423106) at 4°C for 10 minutes to block Fc receptors and assess cell viability. Subsequently, cells were stained with anti-mouse CD45 antibody (Biolegend, 157214) at 4°C for 30 minutes. Cell acquisition and sorting were performed using a Sony MA900 Multi-Application Cell Sorter, with gating strategies applied to exclude doublets and dead cells, followed by the selection of live CD45^+^ leukocytes. Sorted CD45^+^ cells were counted and resuspended at a concentration of 1,000 cells/μL in PBS containing 0.04% BSA. The prepared samples were then submitted to OEbiotech for scRNA-seq.

### ScRNA-seq analysis.

Raw scRNA sequencing reads were aligned to primary DNA sequence of the reference genome (*Mus musculus*, GRCm39 assembly) and the Gencode M33 annotations (Ensembl release 110). The expression matrix is generated with the standard pipeline MobiVision 3.2 (MobiDrop, Zhejiang) of the manufacturer for 3’ droplet-based scRNA-seq. EmptyDrops algorithm is applied to filter out empty droplets.

The expression matrices for each of the 9 samples are quality controlled with the following parameters: (a) each cell should contain at least 1,000 UMIs, (b) each cell should have at least 300 expressing genes, and (c) a percentage of mitochondrial transcript less than 20%. The samples are filtered by these criteria individually before being merged and projected to shared embeddings with CCA integration. A total of 87,942 cells passes the quality filter.

The overall embedding is based on the top 30 PCs from the CCA-integrated dataset, and UMAP is calculated with 25-NN graph. Louvain clustering is performed using 20-NN of the PC space. Cell type annotations are first predicted by SingleR 2.4.1 using the Immunological Genome project transcriptomes as reference (60). This prediction is later refined and proved by classical markers of each cell population. Specifically, γδ T cells are identified by expression of either form of the TCR γ chain gene (*Trgc1-4*). Most of the identified γδ T cells are also detected for *Trdc* expression, since the knockout does not affect the transcription of *Trdc* gene in its 3’ end. Further analysis distinguishes the expression states of γδ T cells from Cd4^+^, Cd8a^+^ αβ T cells, and NK cells. UMAP, clustering, marker identification, and differential gene expression are conducted with the default parameters from the Seurat package.

Enrichment analysis is conducted using the clusterProfiler 4.10.1 package (61). Gene sets are downloaded from the murine part of MSigDB (62), and Ligand-receptor interaction analysis is conducted with CellPhoneDB’s R package, with murine LR pair database adopted from CellChat2 (63, 64). Plotting and statistical testing are conducted with R 4.3.1, pheatmap 1.0.12, Seurat 5.1.0, ktplots 2.4.1 and ggplot2 3.5.1.

### Mouse tumor models and therapeutic effect evaluation.

Subcutaneous tumor–bearing mouse models were successfully established by injecting 1×10^6^ LLC or B16-F10 cells suspended in 100 μL PBS into the right flank of the mice. When the tumor volume reached approximately 50 mm^3^, the mice were randomly allocated into control and radiotherapy groups. For radiotherapy, mice were anesthetized and subcutaneous tumors were irradiated with a single dose of 10 Gy or fractionated doses of 8 Gy×3 or 2 Gy×5. Tumor dimensions, including length (L) and width (W), were measured every other day using a vernier caliper, and tumor volume (V) was calculated using the formula: V = (L × W^2^) / 2. Mice were humanely euthanized when the tumor volume exceeded 1,000 mm^3^. The AKT/MYC-driven orthotopic pancreatic tumor model was generated by Wuhan Moubaili Biotechnology Co. Ltd. For RT-MPs intratumoral injection, postcentrifugation RT-MP precipitates were precisely weighed and reconstituted in PBS at 4 mg/mL. Mice received intratumoral injections of 50 μL PBS or RT-MP suspension every 2 days for 3 total treatments.

### Flow cytometry.

For the analysis of tumor-infiltrating immune cells, fresh tumor tissues were dissociated through mechanical disruption and digestion with Hyaluronidase and Collagenase V. Tissue samples were gently ground through a 40 μm filter into single-cell suspensions, followed by red blood cell (RBC) lysis and resuspension in PBS. To assess cell viability, the single-cell suspensions were incubated with Zombie NIR Fixable Viability Kit (Biolegend, 423106). For the analysis of myeloid cells, the suspensions were incubated at 4°C for 30 minutes with the following antibodies: CD45 (Biolegend, 157208; 157214), CD11b (Biolegend, 101228), F4/80 (Biolegend, 123114; 123116), Ly6C (Biolegend, 128033), Ly6G (Biolegend, 127614), and Gr1 (Biolegend, 108412). For T cell analysis, cells were stained with CD3 (Biolegend, 100204), CD4 (Biolegend, 100422), CD8a (Biolegend, 100752), TCRβ (Biolegend, 109212), TCRγ/δ (Biolegend, 107508), and TCR Vγ4 (BD Pharmingen, 569445). For intracellular cytokine staining, single-cell suspensions were stimulated for 4 hours at 37°C with Monensin sodium salt (ab120499, Abcam, 1 μg/mL), Ionomycin calcium salt (5608212, PeproTech, 100 ng/mL), and Phorbol 12-myristate 13-acetate (PMA) (ab120297, Abcam, 100 ng/mL). Following stimulation, cells were fixed, permeabilized, and stained with IFNγ (Biolegend, 505841; 505830), Granzyme B (Biolegend, 372208), Foxp3 (eBioscience, 17-5773-82), IL-17A (Biolegend, 506922), TNFα (Biolegend, 506341), IL-4 (Biolegend, 504125), IL-10 (Biolegend, 505009), and TGFβ1 (Biolegend, 141407).

### ELISA.

The concentrations of IL-17A in tumor interstitial fluid from mice and peripheral blood plasma from patients were quantified using ELISA kits (DAKEWE, 1211702;1111702), following the protocols provided by the manufacturer.

### Generation of BMDMs.

BMDMs were isolated from the femora of 6- to 12-week-old C57BL/6 mice. Following RBCs lysis, cells were plated and cultured in RPMI-1640 medium supplemented with 10% FBS, 1% penicillin-streptomycin, and recombinant murine M-CSF (20 ng/mL, PeproTech). The culture medium was refreshed every 2 days and on the seventh day, naive BMDMs were harvested for subsequent experiments.

### Isolation of RT-MPs.

To generate RT-MPs, 5 × 10^6^ LLC cells were seeded in 10-cm dishes and exposed to a single dose of 20 Gy or 10 Gy. Following irradiation, the culture medium was replaced. After incubation for 72 hours, the cell medium was collected and centrifuged at 1,000*g* for 10 minutes and then 14,000*g* for 2 minutes to remove tumor cells and debris. The supernatant was then centrifuged again at 14,000*g* at 4°C for 1 hour to pellet the RT-MPs. The precipitate (containing MPs) was washed twice and resuspended in sterile 1× PBS for subsequent experiments.

### dsDNA detection and clearance.

The presence of dsDNA in RT-MPs was quantified using the dsDNA HS Assay Kit (Yeasen, 12640ES60). Fluorescence intensity was measured at excitation/emission wavelengths of 480/520 nm using a fluorescence microplate reader. To eliminate dsDNA, isolated RT-MPs were treated with DNase I (Sigma-Aldrich, 10104159001) following the manufacturer’s protocol.

### Transfections.

For siRNA-mediated knockdown of *Sting* and *P65*, BMDMs were seeded in 6-well plates and transfected with either target-specific siRNA or negative control siRNA using Lipofectamine RNAiMAX Transfection Reagent (Invitrogen, 13778150), in accordance with the manufacturer’s protocol. Cells were harvested 48 hours after transfection, and knockdown efficiency was validated by RT-qPCR and Western blotting. All siRNA primers were custom-synthesized by Sangon Biotech (Shanghai) Co. Ltd. The siRNA sequences used were listed in Supplemental Table 1.

### Macrophage depletion.

For macrophage depletion studies, clodronate liposomes-Anionic (FormuMax, F70101C-A-10) was administered intraperitoneally at a dose of 200 μL per mouse 1 day before radiotherapy. This was followed by subsequent injections of 150 μL per mouse every 2 days for a total of 3 times.

### MDSC depletion.

For MDSC depletion studies, anti-Gr1 (BioXcell, BE0075) was administered intraperitoneally at an initial dose of 200 μg per mouse one day before radiotherapy. Subsequent doses of 100 μg per mouse were injected every 3 days, for a total of 3 times.

### Western blotting.

Cells were lysed using RIPA buffer supplemented with protease and phosphatase inhibitors at 4°C for 30 minutes. The lysates were centrifuged at 12,000*g* for 30 minutes at 4°C, and the supernatant was collected for protein quantification using the BCA protein assay kit (Servicebio, G2026). Protein samples were denatured in SDS-polyacrylamide gel electrophoresis (SDS-PAGE) loading buffer (Servicebio, G2075) by boiling at 100°C for 10 minutes. The proteins were then separated by SDS-PAGE and transferred onto 0.22 μm polyvinylidene difluoride (PVDF) membranes. Membranes were blocked with 5% non-fat milk in Tris-buffered saline containing 0.05% Tween 20 (TBST) for 1 hour at room temperature, followed by incubation with primary antibodies at 4°C overnight. The next day, after washing with TBST, membranes were incubated with HRP-conjugated secondary antibodies at room temperature for 1 hour. Protein bands were visualized using NcmECL Ultra (NCM Biotech, P10100) according to the manufacturer’s instructions. The antibodies used were provided in Supplemental Table 2.

### Real-time quantitative PCR.

Total RNA was isolated using the Total RNA Kit I (Omega, R6834), and RNA concentration was quantified using the NanoDrop ND-1000 spectrophotometer (Thermo Fisher Scientific). Reverse transcription was performed using the HiScript III RT SuperMix (+gDNA wiper) (Vazyme, R323-01) following the manufacturer’s protocol. The resulting complementary DNA (cDNA) was used as the template for quantitative PCR (qPCR) with ChamQ SYBR qPCR Master Mix (Vazyme, Q311-02) on the StepOnePlus Real-Time PCR System (Thermo Fisher Scientific). Gene expression levels were normalized to Glyceraldehyde-3-phosphate dehydrogenase (GAPDH) and analyzed using the comparative threshold cycle (2^–ΔΔCt^) method. All primers used in this study were commercially synthesized by Wuhan GeneCreate Biological Engineering Co. Ltd, and the sequences were listed in Supplemental Table 3.

### ChIP.

BMDMs were cross linked with 1% formaldehyde for 10 minutes at room temperature, followed by washing with PBS. ChIP assays were performed using the ChIP Assay Kit (Beyotime, P2078) according to the manufacturer’s protocol. Anti-NF-κB p65 antibody (A19653) was obtained from ABclonal, and control IgG (A7016) was purchased from Beyotime. The primer sequences were listed in Supplemental Table 4 and were synthesized by Wuhan GeneCreate Biological Engineering Co., Ltd.

### Immunofluorescence staining.

Immunofluorescence staining was conducted on paired peripheral blood tissues obtained from patients with NSCLC who were undergoing radiotherapy. PBMCs were isolated from heparinized blood using density gradient centrifugation, followed by paraffin embedding and sectioning. For immunofluorescence, paraffin-embedded sections were deparaffinized in xylene, rehydrated through a graded ethanol series, and subjected to antigen retrieval by heating in citrate buffer (10 mM, pH 6.0) for 15 minutes in a microwave oven. Sections were incubated overnight at 4°C with primary antibodies targeting TCRδ (Santa Cruz, sc-100289), IL-17A (Proteintech, 26163-1-AP), and γ-H2AX (Servicebio, GB111841). After washing, sections were incubated with Alexa Fluor 594 (Servicebio, GB28303) or 488 (Servicebio, GB25303) dye-conjugated secondary antibodies for 1 hour at room temperature. Nuclei were stained with DAPI (Servicebio, G1012) for 10 minutes at room temperature. Immunofluorescence images were visualized using the confocal fluorescence microscope (Nikon, AX/AX R with NSPARC).

### Statistics.

Statistical analyses were performed using GraphPad Prism 8.0 software. Comparisons between 2 groups were conducted using unpaired 2-tailed Student’s *t* test or paired *t* test, as appropriate. For comparisons involving more than 2 groups, 1-way ANOVA with Tukey’s multiple comparisons test was applied. Survival curves were compared using the log-rank (Mantel-Cox) test, while tumor growth was analyzed by 2-way ANOVA followed by Tukey’s multiple comparison test. Flow cytometry data were analyzed using FlowJo software (version 10.8.1). A *P* value of less than 0.05 was considered significant.

### Study approval.

All mice were raised in compliance with the protocols approved by the Animal Experimentation Ethics Committee of the Huazhong University of Science and Technology (IACUC Number: 4455). The acquisition of peripheral blood samples from patients with NSCLC before and after radiotherapy was approved by the Medical Ethics Committee of Union Hospital, Tongji Medical College of Huazhong University of Science and Technology. All participants signed informed consent prior to the study.

### Data availability.

scRNA-seq data in raw FASTQ format reported in this paper have been deposited in the Genome Sequence Archive in National Genomics Data Center, China National Center for Bioinformation / Beijing Institute of Genomics, Chinese Academy of Sciences (GSA: CRA024537, BioProject: PRJCA038015), which are publicly accessible at https://ngdc.cncb.ac.cn/gsa Processed count matrices are distributed under OMIX https://ngdc.cncb.ac.cn/omix (accession number OMIX009639). All numerical values for the figures are provided in the Supporting Data Values file in the Supplemental Materials. The data that support the findings of this study are available from the corresponding authors upon reasonable request.

## Author contributions

CW and KY conceived and supervised the project. CW and YD designed the experiments. XL, XY, WW, JW, ZY, YS, YH, HZ, YW, ZZ, LW, and FH performed all experiments. All authors analyzed and discussed the data. CW, YD, and XL wrote the paper.

## Funding support

- The National Natural Science Foundation of China (Grant No. 82330085).
- Key R&D Program of Hubei Province (Grant No. 2024BCB051).
- Chinese Society of Clinical Oncology Foundation (Grant No. Y-MSDZD2022-0476).
- Natural Science Foundation of Hubei Province (Grant No. 2025AFB035).
- The Open Research Fund of Hubei Key Laboratory of Precision Radiation Oncology (2024ZLJZFL007).

## Supplementary Material

Supplemental data

Unedited blot and gel images

Supporting data values

## Version 1 10/07/2025

In-Press Preview

## Version 2 12/15/2025

Electronic publication
