# Supplementary material for: IL-17–producing γδ T cells in the tumor microenvironment promote radioresistance in mice
Source: J Clin Invest. 2025 Oct 7;135(24):e193945. doi: 10.1172/JCI193945 (PMC12700543; doi:10.1172/JCI193945)
Supplement: Supplemental data [file jci-135-193945-s020.pdf]

Supplemental material

IL-17-producing γδ T cells in the tumor microenvironment promote radioresistance in mice

Yue Deng[1,2,3,4†], Xixi Liu[1,2,3,4†], Xiao Yang[1,2,3,4], Wenwen Wei[1,2,3,4], Jiacheng Wang[1,2,3,4],Zheng Yang[1,2,3,4], Yajie Sun[1,2,3,4], Yan Hu[1,2,3,4], Haibo Zhang[5], Yijun Wang[1,2,3,4], Zhanjie Zhang[1,2,3,4], Lu Wen[1,2,3,4], Fang Huang[1,2,3,4], Kunyu Yang[1,2,3,4*], Chao Wan[1,2,3,4*]

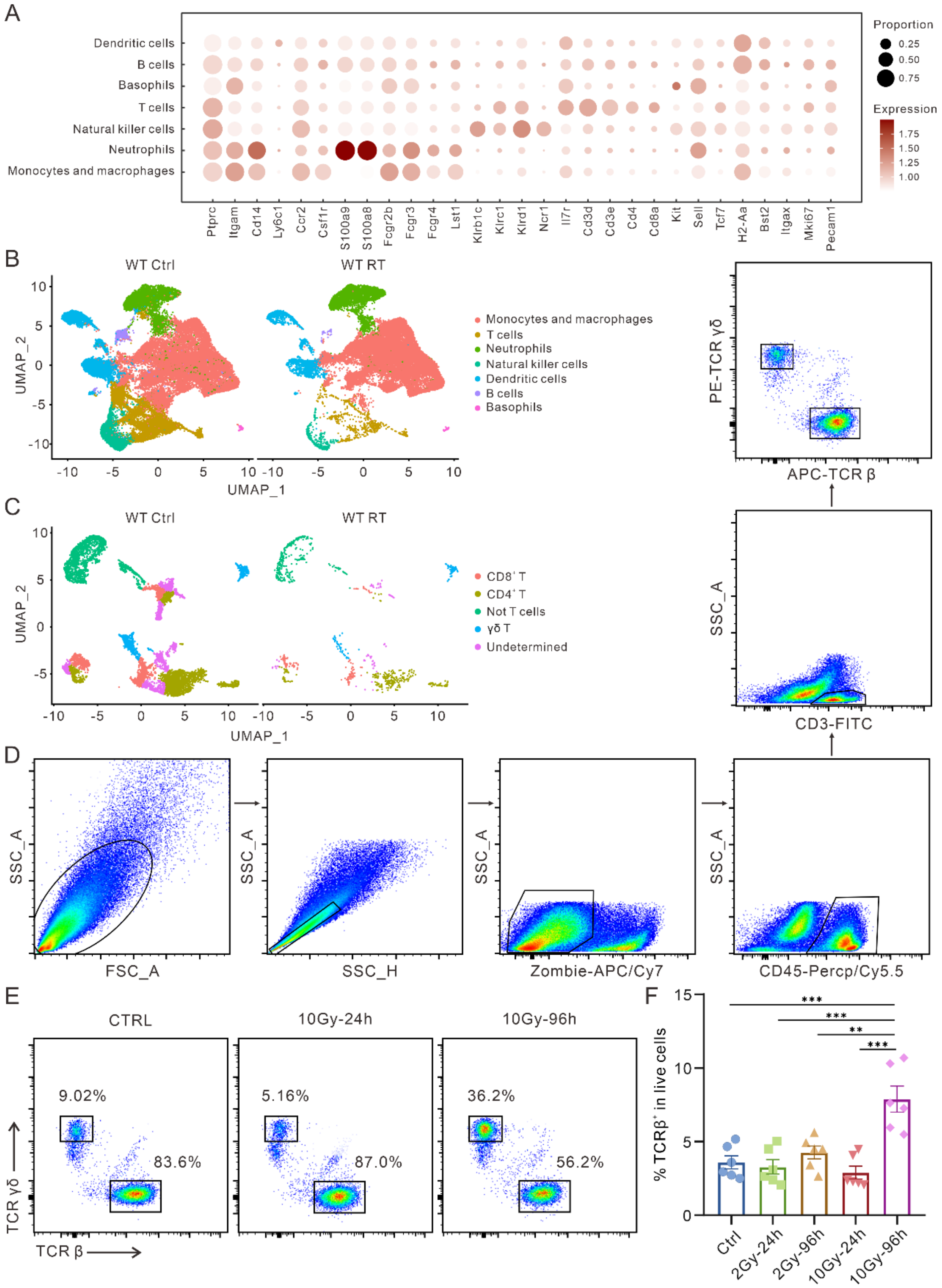

Supplemental Figure 1. Increased γδ T cell infiltration in the TME after radiotherapy. (**A**) Dot plot showing the mean expression of canonical marker genes for seven major cell lineages identified by scRNA-seq. (**B**) UMAP plot of all cells from WT Ctrl and WT RT groups colored by cell identities. (**C**) UMAP plot of T cells from WT Ctrl and WT RT groups colored by cell clusters as indicated. (**D**) Flow cytometry gating strategy for identifying αβ T cells and γδ T cells. (**E**) Representative flow cytometry dot plots showing the percentages of αβ T cells and γδ T cells in the corresponding groups. (**F**) Flow cytometry analysis of αβ T cell proportions in the TME of

LLC subcutaneous tumors following radiotherapy at different doses (2 Gy and 10 Gy) and time points (24h and 96h) (n = 6 per group). $^{**}p < 0.01$; $^{***}p < 0.001$. One-way ANOVA with Tukey's multiple comparisons test (F).

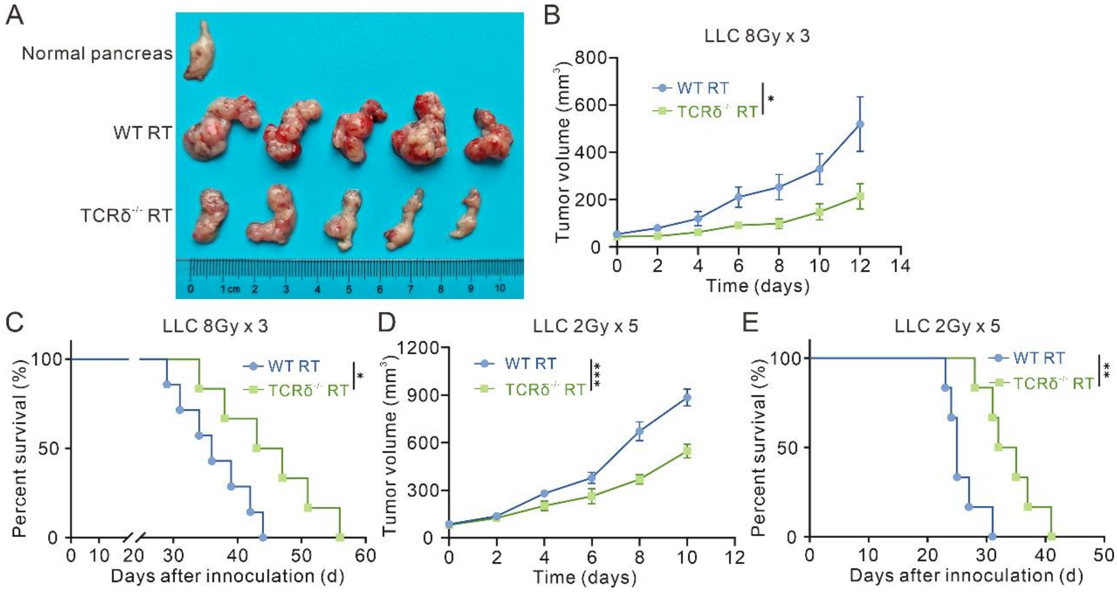

Supplemental Figure 2. γδ T cells drive resistance to radiotherapy. (**A**) Representative images of orthotopic pancreatic tumors and normal pancreas in the AKT/MYC-driven orthotopic model (n = 5 per group). (**B**) Tumor growth curves of LLC subcutaneous tumors following 8 Gy × 3 fractionated radiotherapy (n = 6 per group). (**C**) Kaplan-Meier survival plot of LLC lung cancer-bearing mice following 8 Gy × 3 radiotherapy (n = 6 to 7 per group). (**D**) Tumor growth curves of LLC subcutaneous tumors following 2 Gy × 5 fractionated radiotherapy (n = 5 to 6 per group). (**E**) Kaplan-Meier survival plot of LLC lung cancer-bearing mice following 2 Gy × 5 radiotherapy (n = 6 per group). *$p < 0.05$; **$p < 0.01$; ***$p < 0.001$. Two-way ANOVA (B, D), Log-rank (Mantel-Cox) test (C, E).

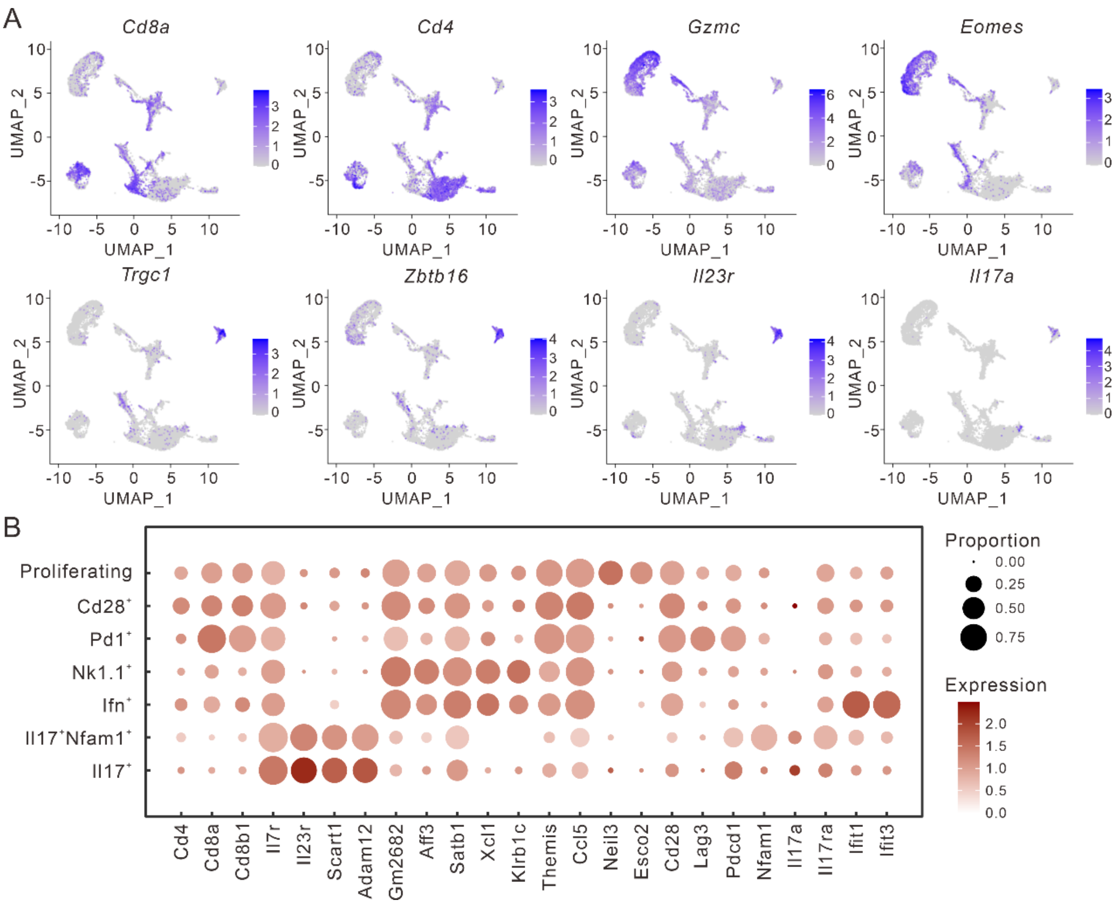

Supplemental Figure 3. ScRNA-seq reveals the characteristics of T cells in the TME. (A) Feature plots depicting the expression of T cell characteristic genes in the major T cell clusters. (**B**) Dot plot showing the mean expression of canonical marker genes for seven major γδ T cell clusters identified by scRNA-seq.

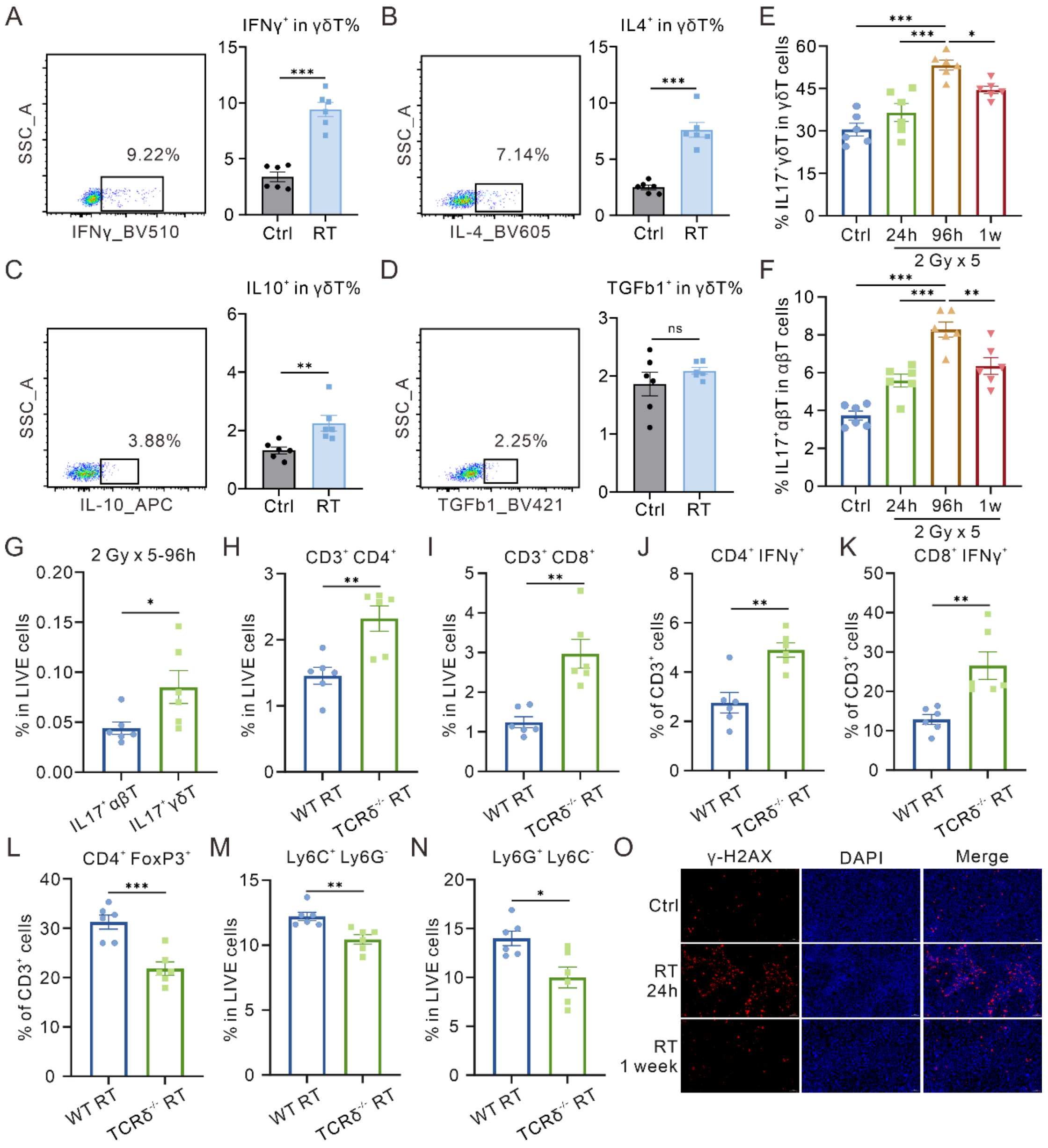

Supplemental Figure 4. Characteristics of γδ T cells in the TME. (**A-D**) Representative flow cytometry plots and statistical analysis of IFN-γ (A), IL-4 (B), IL-10 (C), and TGF-β (D) expression in γδ T cells from LLC subcutaneous tumors after radiotherapy (n = 6 per group). (**E**) Flow cytometry analysis of IL-17^{+} γδ T cell proportions in the TME of LLC subcutaneous tumors following 2 Gy × 5 fractionated radiotherapy at different time points (n = 6 per group). (**F**) Flow cytometry analysis of IL-17^{+} αβ T cell proportions in the TME of LLC subcutaneous tumors following 2 Gy × 5 fractionated radiotherapy at different time points (n = 6 per group). (**G**) Proportions of IL-17^{+} γδ T cells and IL-17^{+} αβ T cells in the TME at 96 hours after 2 Gy × 5 fractionated radiotherapy (n = 6 per group). (**H-N**) Flow cytometry analysis of CD3^{+}CD4^{+} T cell (H), CD3^{+}CD8^{+} T cell (I), CD4^{+}IFNγ^{+} Th1 cell (J), CD8^{+}IFNγ^{+} T cell (K), CD4^{+}FoxP3^{+} Treg cell (L), Ly6C^{+}Ly6G^{-} M-MDSC (M), and Ly6G^{+}Ly6C^{-} PMN-MDSC (N) proportions in the TME of LLC subcutaneous tumors at 1 week post-radiotherapy (n = 6 per group). (**O**) Representative γ-H2AX immunofluorescence staining of LLC subcutaneous tumor sections in corresponding

groups. *$p < 0.05$; **$p < 0.01$; ***$p < 0.001$; ns, not statistically significant. Unpaired two-tailed Student's t-test (A-D, G-N), One-way ANOVA with Tukey's multiple comparisons test (E-F).

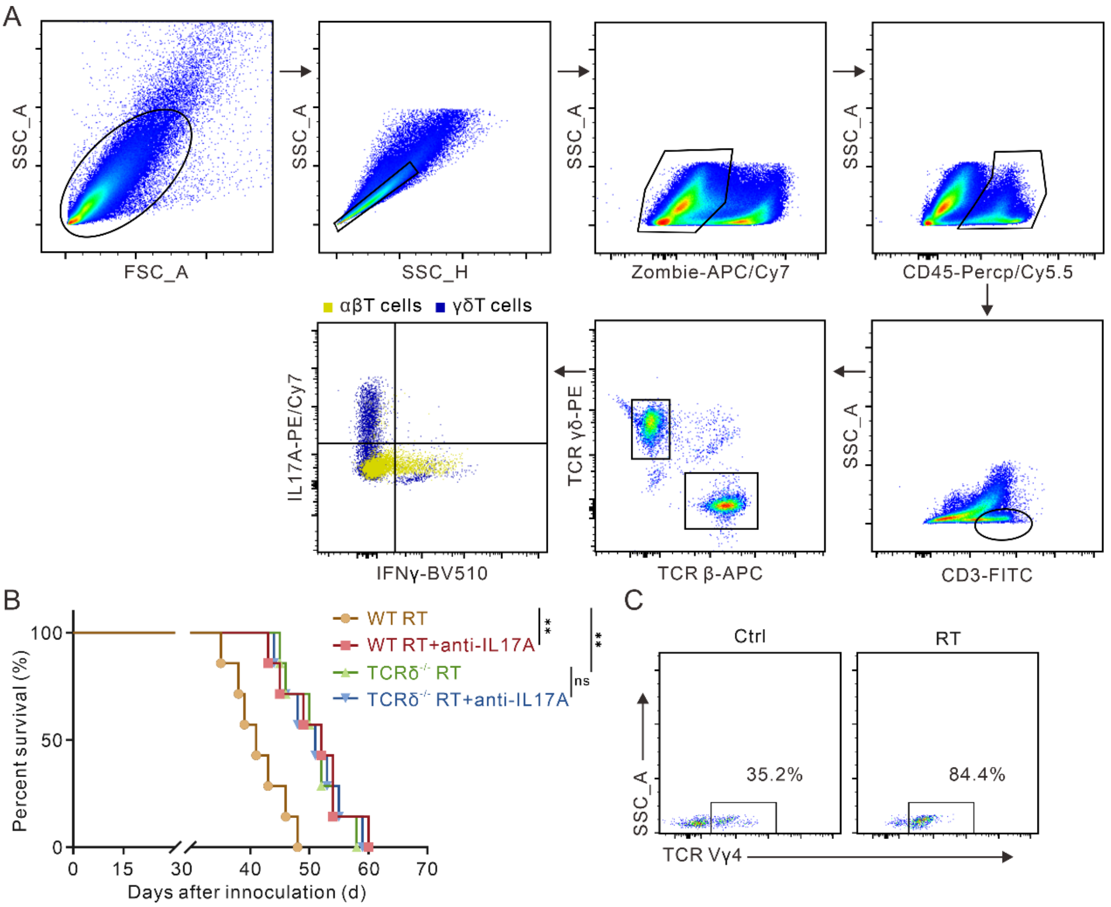

Supplemental Figure 5. γδ T cells are the principal cellular source of IL-17A in the TME. (**A**) Flow cytometry gating strategy for identifying cytokine production in αβ T cells and γδ T cells. (**B**) Kaplan-Meier survival plot of LLC lung cancer-bearing mice in corresponding groups. (n = 7 per group). (**C**) Representative flow cytometry plots of TCR Vγ4^{+} γδ T cell proportions of LLC subcutaneous tumors after radiotherapy. ***p* < 0.01; ns, not statistically significant. Log-rank (Mantel-Cox) test (B).

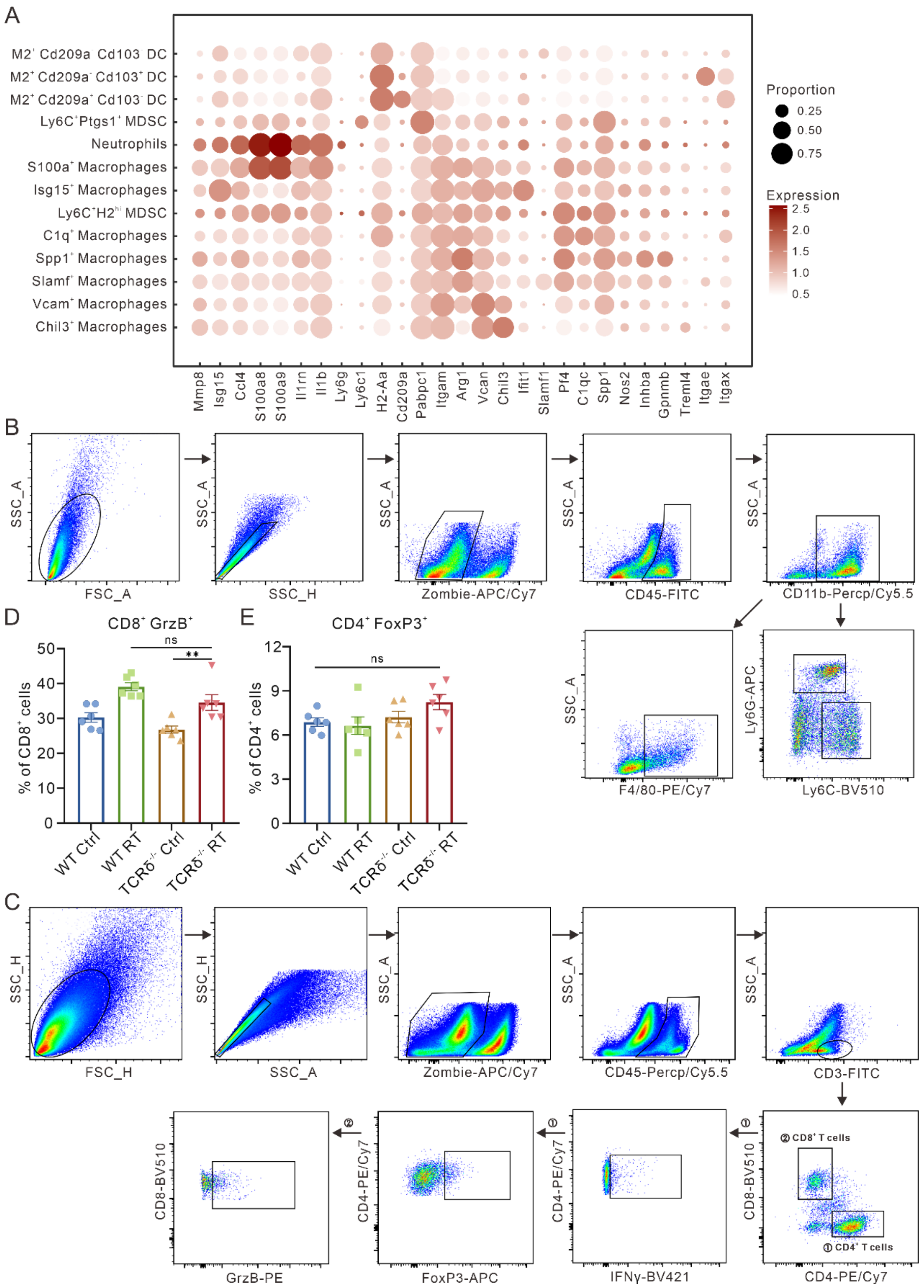

Supplemental Figure 6. γδ T cells in the TME promote MDSCs recruitment and impair T cell activation. (**A**) Dot plot showing the mean expression of canonical monocytes and macrophages marker genes for major thirteen cell lineages identified by scRNA-seq. (**B**) Flow cytometry gating strategy for identifying myeloid cells. (**C**) Flow cytometry gating strategy for identifying CD4^{+} T cells and CD8^{+} T cells. (**D-E**) Flow cytometry analysis of CD8^{+}GrzB^{+} T cell (D) and CD4^{+}FoxP3^{+} T cell (E) proportions in the TME of LLC subcutaneous tumors in

corresponding groups (n = 6 per group). $**p < 0.01$; ns, not statistically significant. One-way ANOVA with Tukey's multiple comparisons test (D-E).

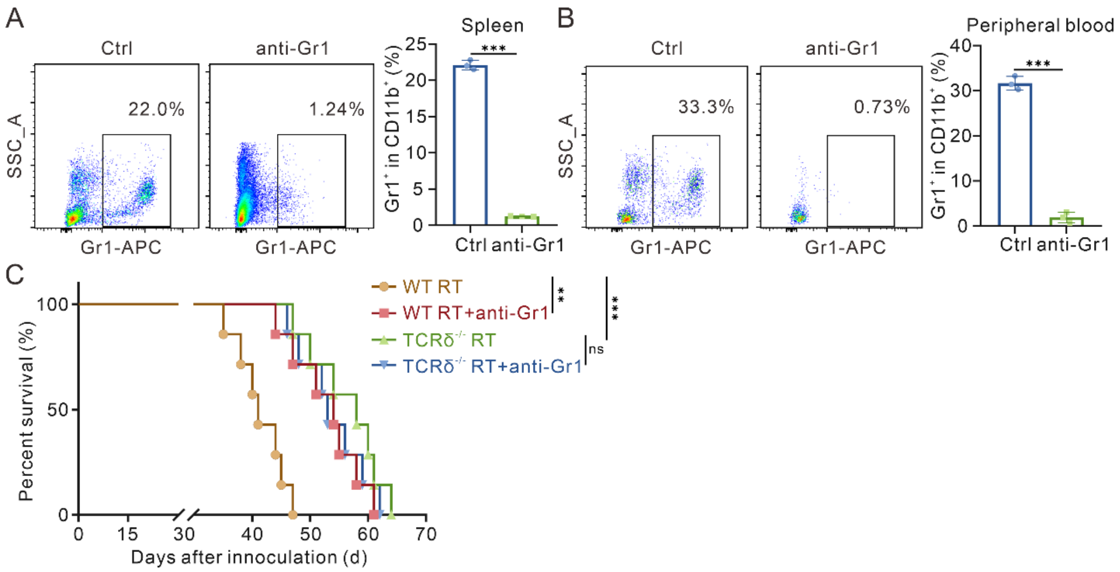

Supplemental Figure 7. γδ T cells drive radioresistance via MDSC-mediated immunosuppression. (**A**) MDSCs clearance efficiency in mouse spleen detected by flow cytometry (n = 3 per group). (**B**) MDSCs clearance efficiency in mouse peripheral blood detected by flow cytometry (n = 3 per group). (**C**) Kaplan-Meier survival plot of LLC lung cancer-bearing mice in corresponding groups. (n = 7 per group). ***p* < 0.01; ****p* < 0.001; ns, not statistically significant. Unpaired two-tailed Student’s t-test (A-B), Log-rank (Mantel-Cox) test (C).

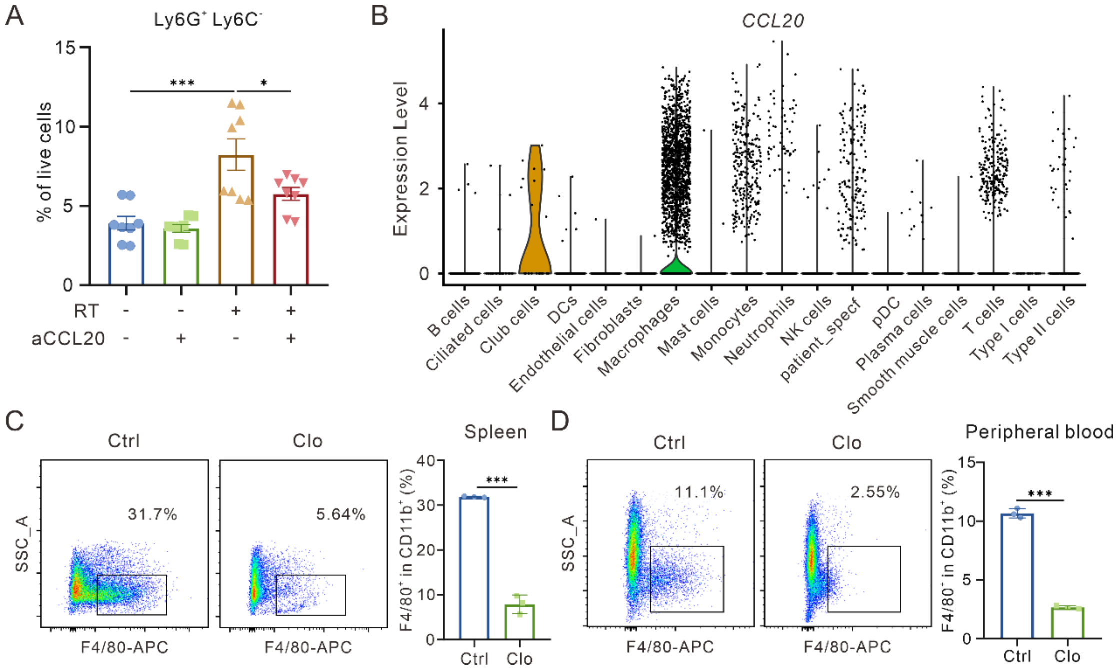

Supplemental Figure 8. Macrophages are the primary source of the chemokine CCL20. (**A**) Flow cytometry analysis of Ly6G^{+}Ly6C^{-} PMN-MDSC proportions in the TME of LLC subcutaneous tumors in corresponding groups (n = 8 per group). (**B**) Violin plots demonstrate the *CCL20* expression in the major cell clusters. (**C**) Macrophages clearance efficiency in mouse spleen detected by flow cytometry (n = 3 per group). (**D**) Macrophage clearance efficiency in mouse peripheral blood detected by flow cytometry (n = 3 per group). **p* < 0.05; ****p* < 0.001. One-way ANOVA with Tukey's multiple comparisons test (A), Unpaired two-tailed Student's t-test (C-D).

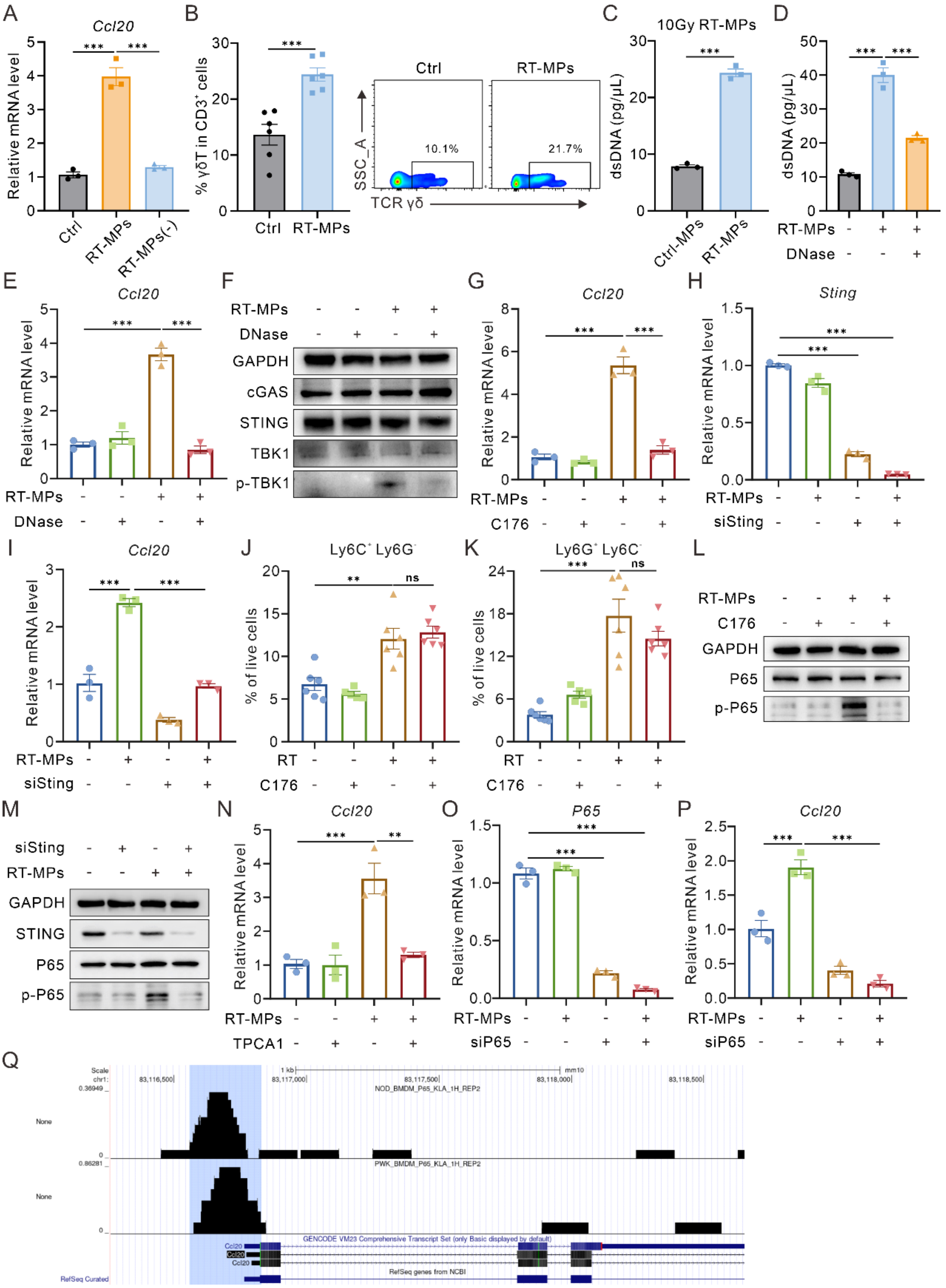

Supplemental Figure 9. RT-MPs transcriptionally upregulate *Ccl20* expression in macrophages via the cGAS-STING/NF-κB pathway. (**A**) Relative mRNA expression of *Ccl20* in BMDMs treated with 10 Gy-induced RT-MPs or irradiated tumor cell-derived CM depleted of RT-MPs. (**B**) Flow cytometry analysis of γδ T cell proportions in the TME of LLC subcutaneous tumors after intratumoral injection of RT-MPs (n = 6 per group). (**C**) Quantitative measurement of dsDNA content in 10 Gy-induced RT-MPs and Ctrl-MPs. (**D**) Quantitative measurement of dsDNA content in RT-MPs treated with DNase. (**E**) Relative mRNA expression of *Ccl20* in

BMDMs treated with 10 Gy-induced RT-MPs or dsDNA-depleted RT-MPs. (**F**) Representative Western blot images showing protein expression levels of cGAS, STING, TBK1 and p-TBK1 in BMDMs treated with 10 Gy-induced RT-MPs or dsDNA-depleted RT-MPs. (**G**) Relative mRNA expression of *Ccl20* in BMDMs treated with 10 Gy-induced RT-MPs or the STING inhibitor C176. (**H**) Relative mRNA expression of *Sting* in BMDMs transfected with STING-targeting siRNAs. (**I**) Relative mRNA expression of *Ccl20* in BMDMs treated with RT-MPs or STING-targeting siRNAs. (**J-K**) Flow cytometry analysis of $Ly6C^{+}Ly6G^{-}$ M-MDSC (J), $Ly6G^{+}Ly6C^{-}$ PMN-MDSC (K) proportions in the TME of LLC subcutaneous tumors in corresponding groups (n = 5 to 6 per group). (**L**) Representative Western blot images showing protein expression levels of P65 and p-P65 in BMDMs treated with 10 Gy-induced RT-MPs or C176. (**M**) Representative Western blot images showing protein expression levels of STING, P65 and p-P65 in BMDMs treated with RT-MPs or STING-targeting siRNAs. (**N**) Relative mRNA expression of *Ccl20* in BMDMs treated with 10 Gy-induced RT-MPs or the NF-κB pathway inhibitor TPCA1. (**O**) Relative mRNA expression of *P65* in BMDMs transfected with P65-targeting siRNAs. (**P**) Relative mRNA expression of *Ccl20* in BMDMs treated with RT-MPs or P65-targeting siRNAs. (**Q**) UCSC Genome Browser tracks displaying the *Ccl20* promoter region with P65 ChIP-seq data from ENCODE. **$p < 0.01$; ***$p < 0.001$; ns, not statistically significant. One-way ANOVA with Tukey's multiple comparisons test (A, D-E, G-K, N-P), Unpaired two-tailed Student's t-test (B-C).

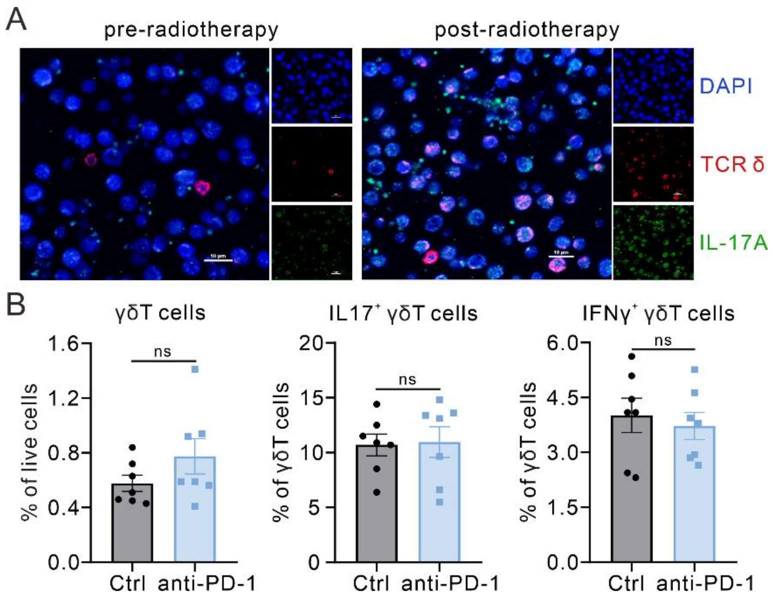

Supplemental Figure 10. γδ T cell depletion does not enhance the efficacy of immunotherapy. (**A**) Representative immunofluorescence staining of γδ T cell marker TCRδ (red) and IL-17A (green) in peripheral blood PBMCs from lung cancer patients pre- and post-radiotherapy. Scale bar: 10 µm. (**B**) Flow cytometry analysis of γδ T cell, IL-17^{+} γδ T cell, and IFN-γ^{+} γδ T cell proportions in the TME of LLC subcutaneous tumors after immunotherapy (n = 7 per group). ns, not statistically significant. Unpaired two-tailed Student's t-test (B).

Supplemental Table 1. Sequences of designed siRNA targeting *Sting* and *P65*.

siRNA	Sense Seq (5'-3')	Antisense Seq (5'-3')
Sting	GCCUCAUUGUCUACCAAGA	UCUUGGUAGACAAUGAGGC
P65	GCAGUUUGAUGCUGAUGAA	UUCAUCAGCAUCAAACUGC

Supplemental Table 2. Antibodies used in the Western blotting.

Antibodies	Source	Detailed information
GAPDH	ABclonal	GAPDH Rabbit pAb (AC001)
cGAS	Proteintech	cGAS Polyclonal antibody (29958-1-AP)
STING	Proteintech	STING Polyclonal antibody (19851-1-AP)
TBK1	Proteintech	TBK1 Polyclonal antibody (28397-1-AP)
Phospho-TBK1	CellSignalingTechnology	Phospho-TBK1/NAK (Ser172) Rabbit mAb (5483)
P65	ABclonal	NF-κB p65/RelA Rabbit mAb (A19653)
Phospho-P65	Proteintech	Phospho-NF-κB p65 (Ser468) Recombinant antibody (82335-1-RR)
Second antibody	ABclonal	HRP Goat Anti-Rabbit IgG (H+L) (AS014)

Supplemental Table 3. Sequences of primers for RT-qPCR.

Gene name	Forward Primer (5’-3’)	Reverse Primer (5’-3’)
Gapdh (m)	TGACCTCAACTACATGGTCTACA	CTTCCCATTCTCGGCCTTG
GAPDH (h)	CTGGGCTACACTGAGCACC	AAGTGGTCGTTGAGGGCAATG
Ccl20 (m)	AGGCAGAAGCAAGCAACTACGAC	ATCGGCCATCTGTCTTGTGAAACC
Sting (m)	CCAAGAACCCACAGACGGAAACAG	GGAGGAGGTGCCACTGAGGTC
P65 (m)	AGGCTTCTGGGCCTTATGTG	TGCTTCTCTCGCCAGGAATAC
Ccl2 (m)	CACTCACCTGCTGCTACTCATTCAC	CTTCTTTGGGACACCTGCTGCTG
Ccl3 (m)	CTCCCAGCCAGGTGTCATTTTCC	CAGGCATTCAGTTCCAGGTCAGTG
Ccl4 (m)	TTGCTCGTGGCTGCCTTCTG	GAAGCTGCCGGGAGGTGTAAG
Ccl5 (m)	GACACCACTCCCTGCTGCTTTG	CTCTGGGTTGGCACACACTTGG
Ccl8 (m)	GCTCCAGTCACCTGCTGCTTTC	ACACAGAGAGACATACCCTGCTTGG
Ccl17 (m)	GGTCACTTCAGATGCTGCTCCTG	CCTGGACAGTCAGAAACACGATGG
Ccl19 (m)	TGCTAATGATGCGGAAGACTGCTG	TCCTTCTGGTGCTGTTGCCTTTG
Ccl21a (m)	GAGCCTCCTTAGCCTGGTCCTG	TGTTCAGTTCTCTTGCAGCCCTTG
Ccl22 (m)	CTCCTGGTGGCTCTCGTCCTTC	CGGCACAGATATCTCGGTTCTTGAC
Ccl25 (m)	TTTGAAGACTGCTGCCTGGGTTAC	CACTCCTCACGCTTGTACTGTTGG
Ccl27 (m)	CTACCGAAAGCCACTCTCAGACAAG	TCAGCCCATTTTCCTTAGCATCCC
Ccl27a (m)	CTGGCATCCGTGGAACAAGACTAAG	GTCCCTTGGAGCCTTTTCCCTTG
Ccl28 (m)	GTGTGTGTGGCTTTTCAAACCTCAG	AAGTACGATTGTGCGGGCTGATG
Cxcl2 (m)	AACATCCAGAGCTTGAGTGTGACG	GGGCTTCAGGGTCAAGGCAAAC
Cxcl5 (m)	TGCGTTGTGTTTGCTTAACCGTAAC	TGACTTCCACCGTAGGGCACTG
Cxcl8 (m)	TCGGGAGACCTCTAGACACTTTGC	GCCTGTCAAGCTGACTTCACTGG
Cxcl9 (m)	TCCTTTTGGGCATCATCTTCC	TTTGTAGTGGATCGTGCCTCG
Cxcl10 (m)	CCAAGTGCTGCCGTCATTTTC	GGCTCGCAGGGATGATTTCAA
Tcrvg4 (m)	CTTGCAACCCCTACCCATAT	AAATGTCTGCATCAAGTCT
TRDV2 (h)	GCCATTGAGTTGGTGCCTGA	GGATGGTTTGGTATGAGGCTG
TRGV9 (h)	GCAGGTCACCTAGAGCAACC	GAAGGAAGAAAAATAGTGGGC

Supplemental Table 4. Sequences of primers targeting *Ccl20* for ChIP.

	Forward Primer (5’-3’)	Reverse Primer (5’-3’)
Primer 1	CAGGAAGTTTTCCCTGTGGGT	GGGAATGTACACAAGAAGGCG
Primer 2	TTTGTGGTGACAGGATGAGGC	TCCAGCACCAGCCCTTCTTAT
