# Supplementary material for: IL-17–producing γδ T cells in the tumor microenvironment promote radioresistance in mice
Source: J Clin Invest. 2025 Oct 7;135(24):e193945. doi: 10.1172/JCI193945 (PMC12700543; doi:10.1172/JCI193945)

Figure 5H:

GAPDH:

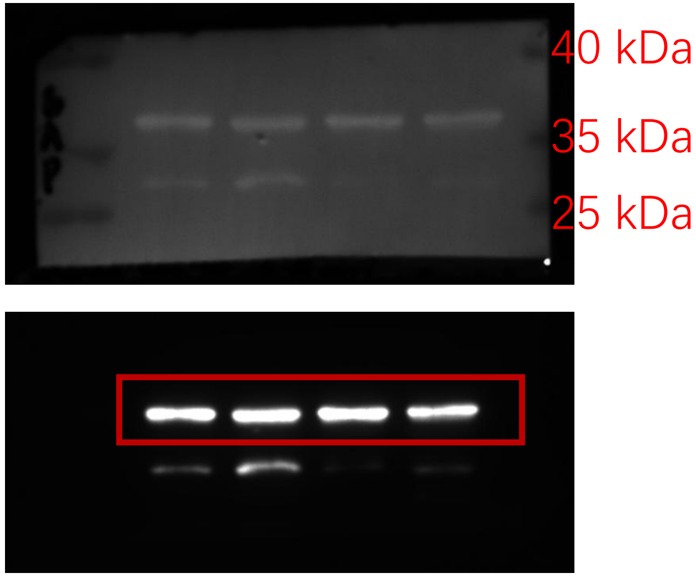

cGAS:

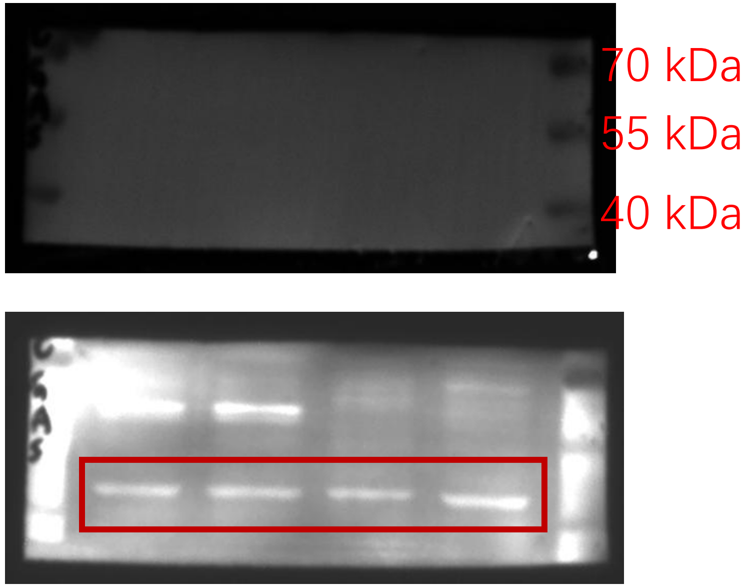

STING:

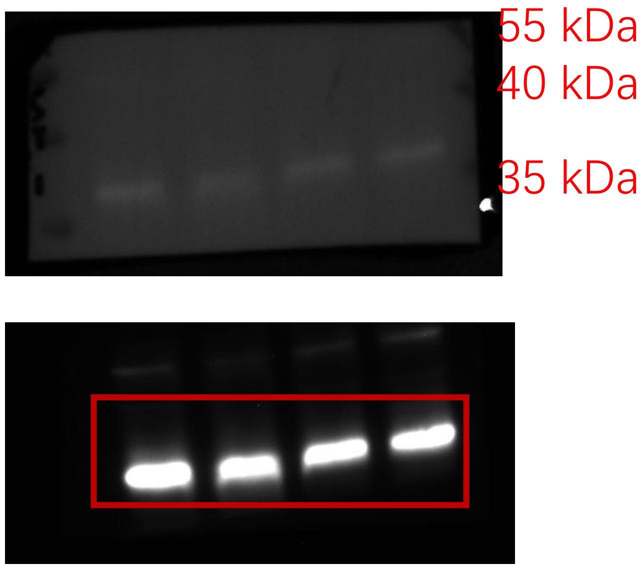

Figure 5H:

TBK1:

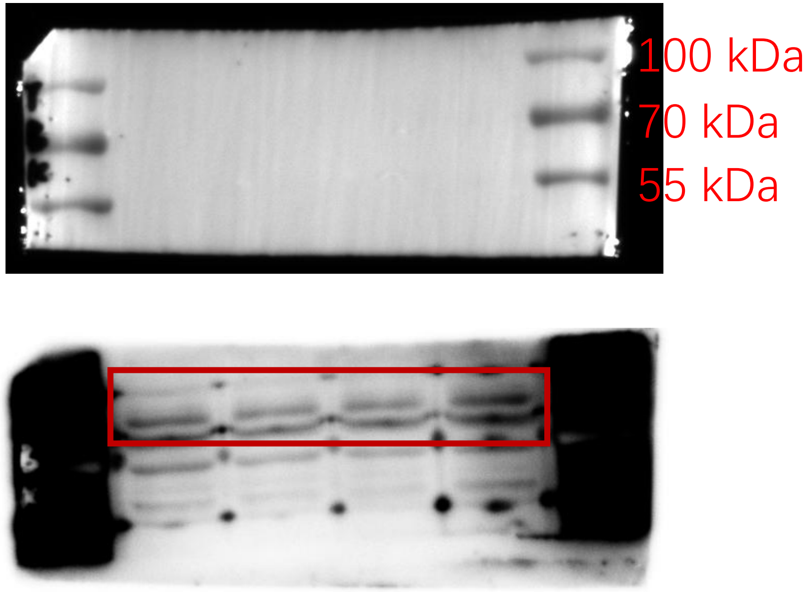

p-TBK1:

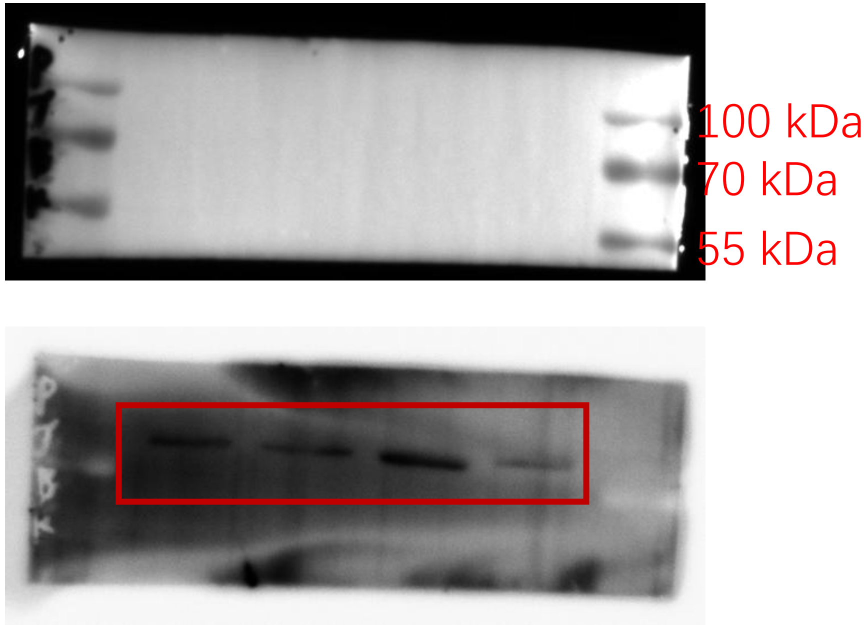

Figure 5K:

GAPDH:

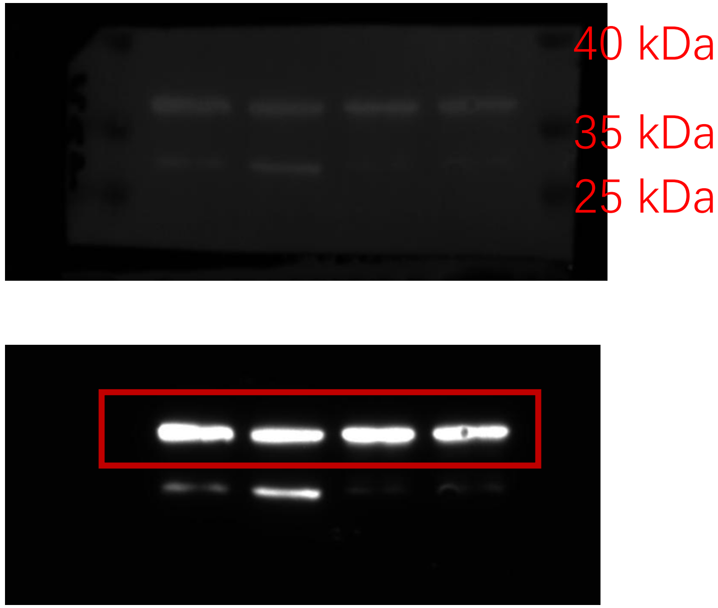

P65:

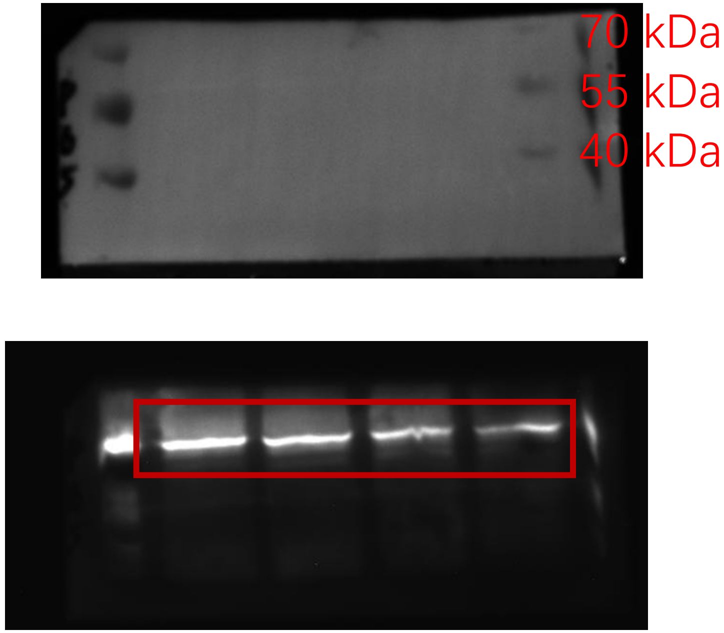

p-P65:

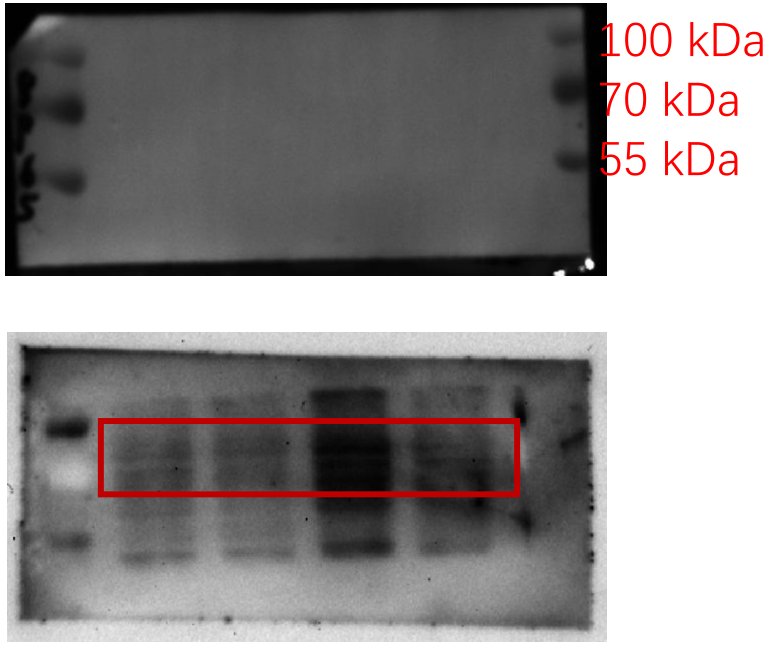

Figure 5M:

primer1:

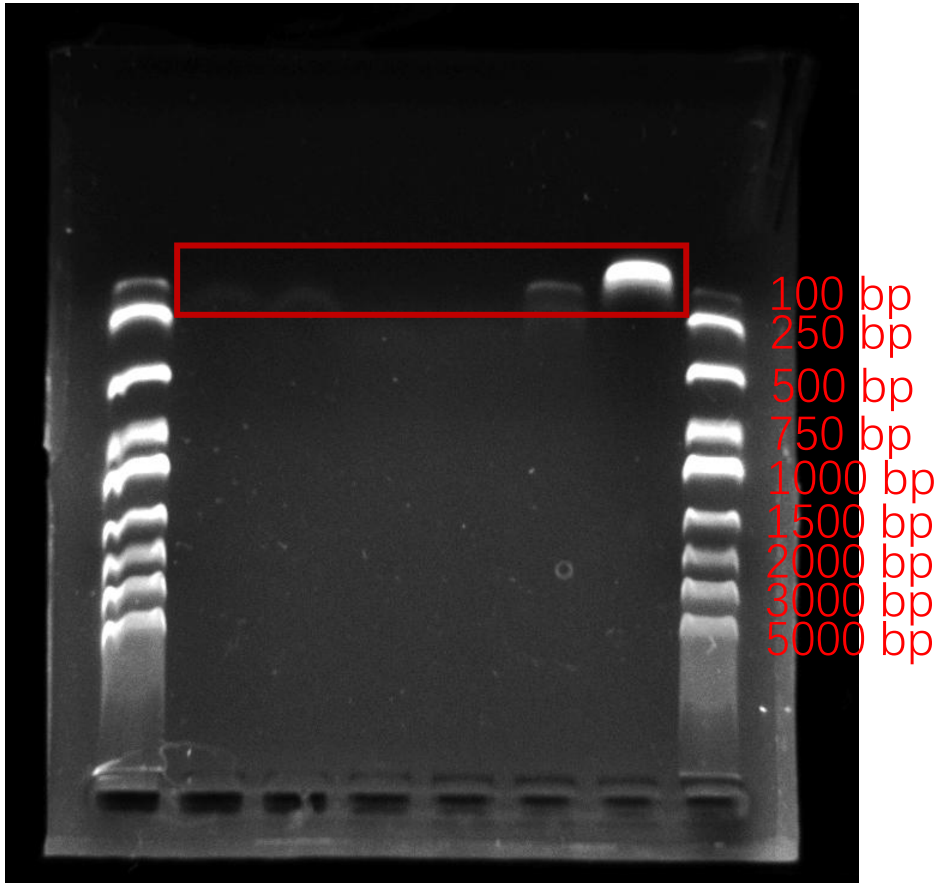

primer2:

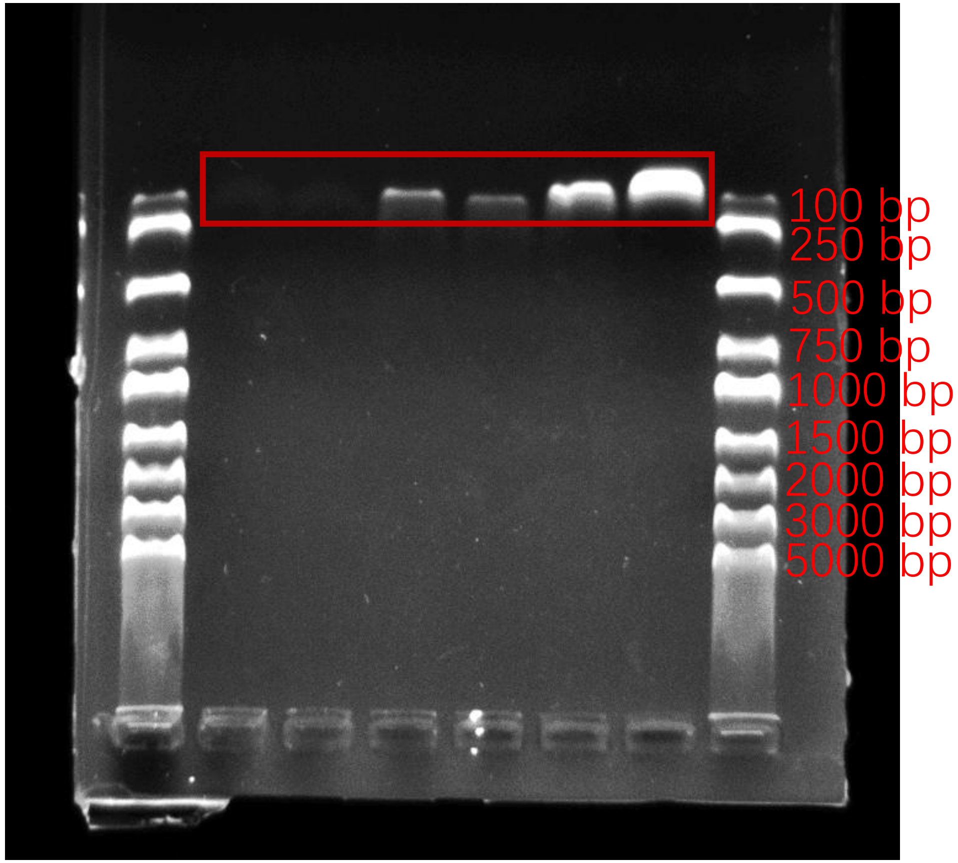

Figure S9F:

GAPDH:

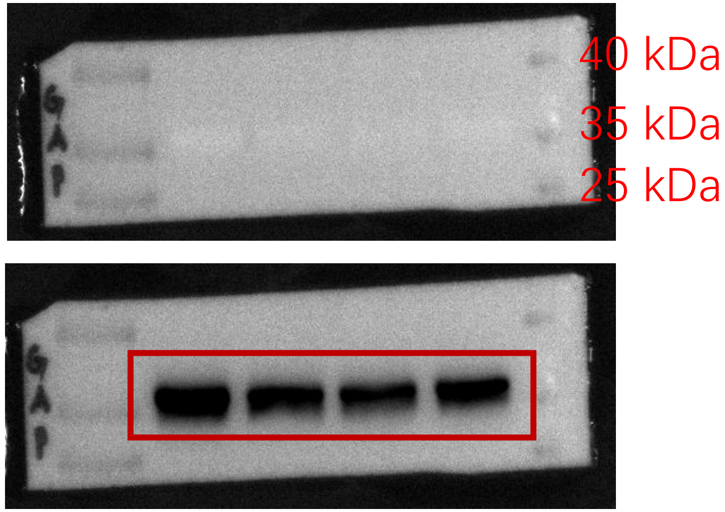

cGAS:

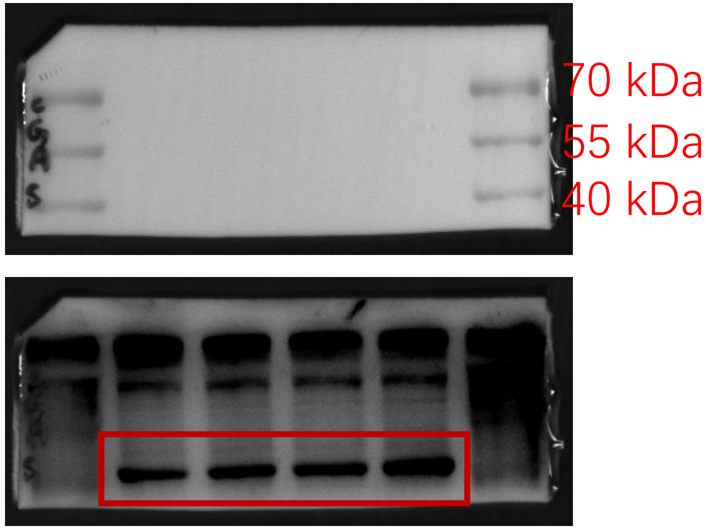

STING:

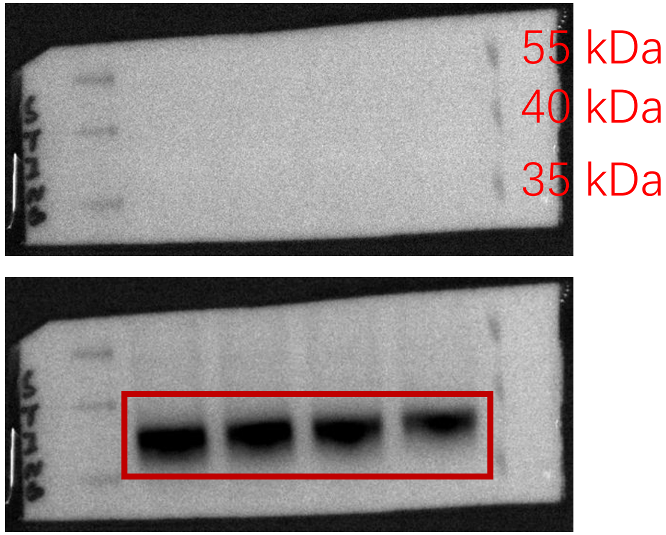

Figure S9F:

TBK1:

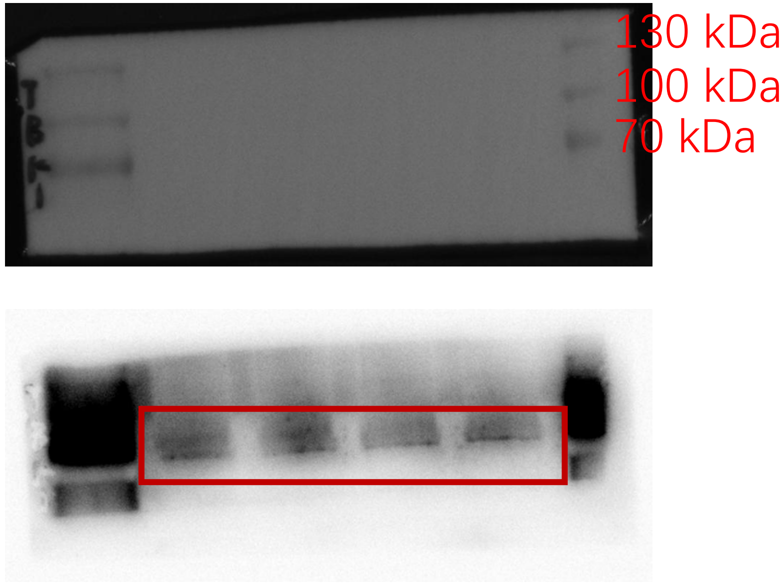

p-TBK1:

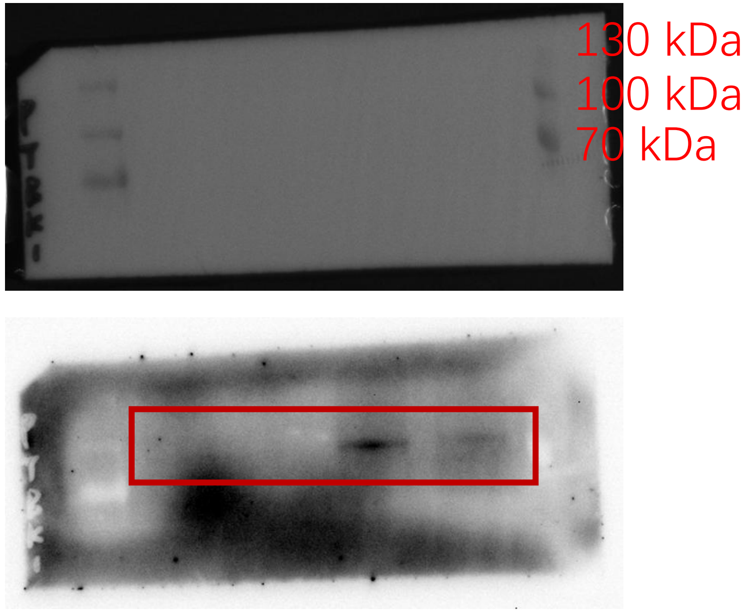

Figure S9L:

GAPDH:

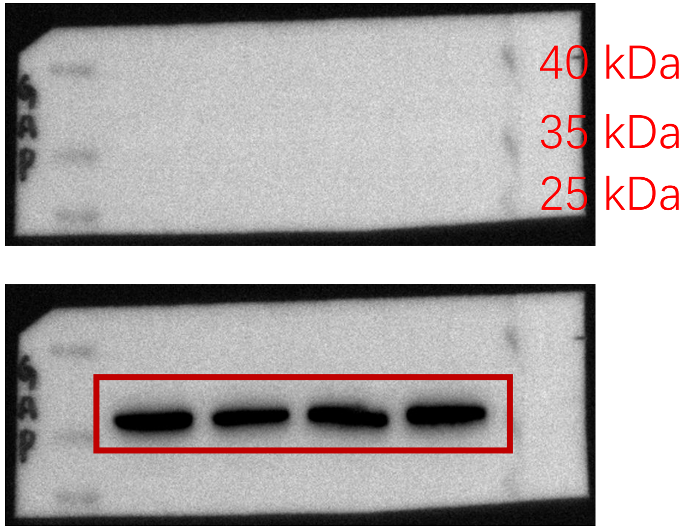

P65:

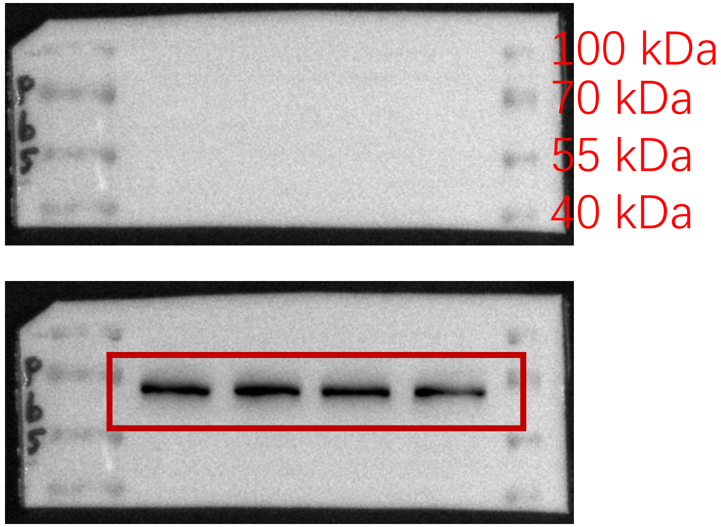

p-P65:

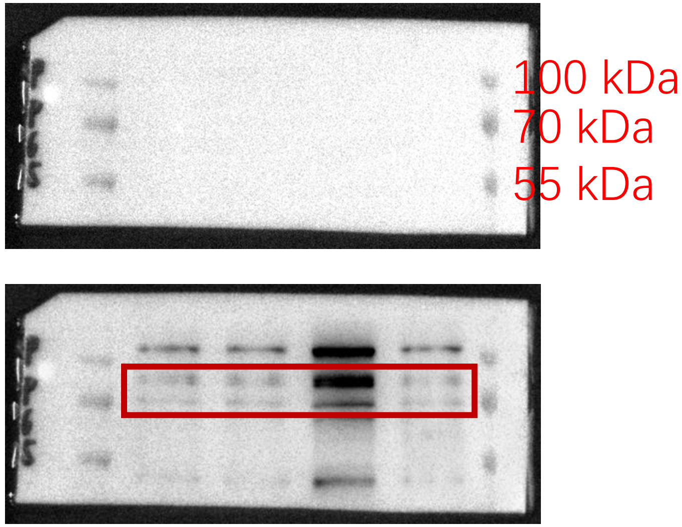

Figure S9M:

GAPDH:

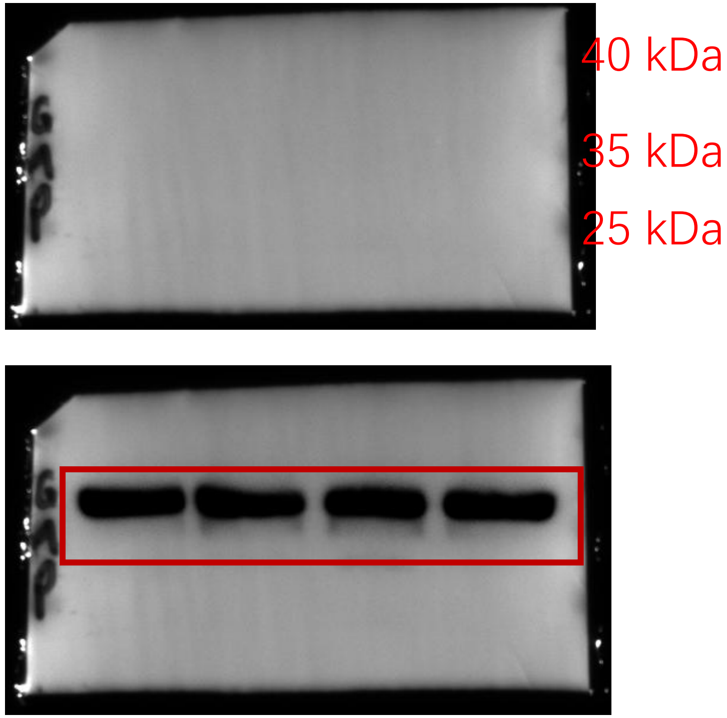

STING:

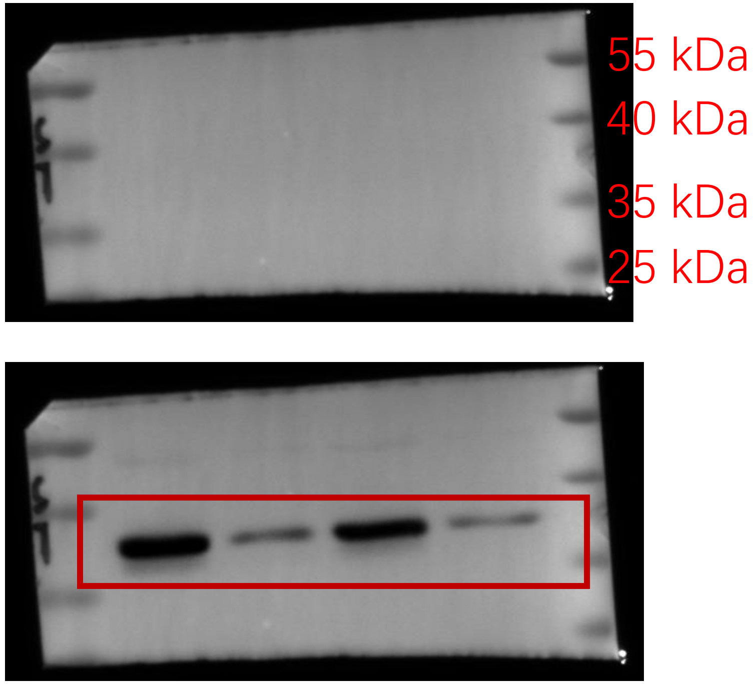

P65:

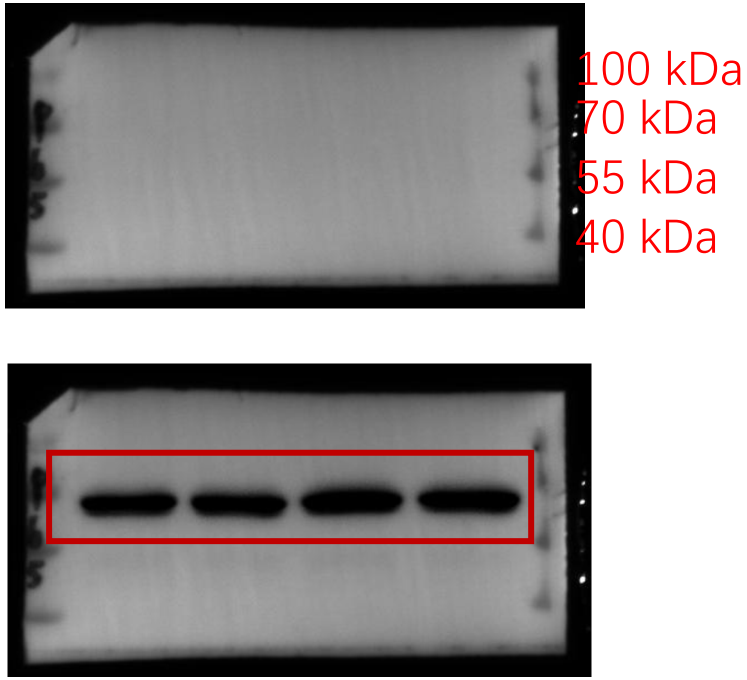

p-P65:

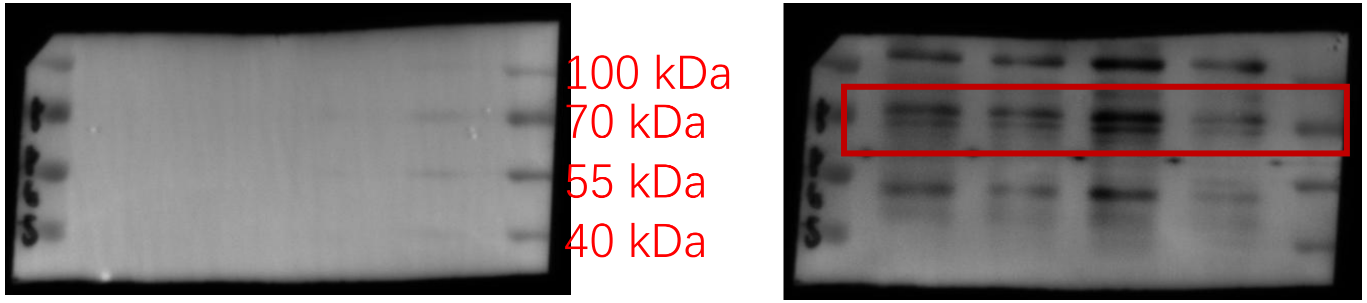

Supplement: Unedited blot and gel images [file jci-135-193945-s021.pdf]
